# Rapid generation of high-quality structure figures for publication with PyMOL-PUB

**DOI:** 10.1093/bioinformatics/btae139

**Published:** 2024-03-06

**Authors:** Yuting Chen, Haoling Zhang, Wen Wang, Yue Shen, Zhi Ping

**Affiliations:** College of Life Sciences, University of Chinese Academy of Sciences, Beijing 100049, China; BGI Research, Shenzhen 518083, China; BGI Research, Shenzhen 518083, China; BGI Research, Shenzhen 518083, China; BGI Research, Changzhou 213299, China; BGI Research, Shenzhen 518083, China; BGI Research, Changzhou 213299, China; BGI Research, Shenzhen 518083, China; BGI Research, Changzhou 213299, China; School of Medicine, The Chinese University of Hong Kong, Shenzhen 518172, China

## Abstract

**Motivation:**

The advancement of structural biology has increased the requirements for researchers to quickly and efficiently visualize molecular structures *in silico*. Meanwhile, it is also time-consuming for structural biologists to create publication-standard figures, as no useful tools can directly generate figures from structure data. Although manual editing can ensure that figures meet the standards required for publication, it requires a deep understanding of software operations and/or program call commands. Therefore, providing interfaces based on established software instead of manual editing becomes a significant necessity.

**Results:**

We developed PyMOL-PUB, based on the original design of PyMOL, to effectively create publication-quality figures from molecular structure data. It provides functions including structural alignment methods, functional coloring schemes, conformation adjustments, and layout plotting strategies. These functions allow users to easily generate high-quality figures, demonstrate structural differences, illustrate inter-molecular interactions, and predict performances of biomacromolecules.

**Availability and implementation:**

Our tool is publicly available at https://github.com/BGI-SynBio/PyMOL-PUB.

## 1 Introduction

The function of biological molecules is directly correlated to their structures. Deep understanding and investigation of these structures help researchers discover more about how they work in the life cycle. In particular, as structure determination technologies like electron microscopy (Yip *et al.* 2020), X-ray crystallography (Maveyraud and Mourey 2020), and magnetic resonance imaging (Sekhar and Kay 2019) improve, molecule structure determination becomes more precise and detailed. At present, a technical difficulty has gradually shifted to how to display the achieved molecular structures more comprehensively.

To fulfill the growing requirements for molecular structure visualization, several well-established tools have been developed, such as VMD (Humphrey *et al.* 1996), PyMOL (DeLano 2002), and ChimeraX (Pettersen *et al.* 2004). However, structure data requires specific visual modifications to produce publication-quality figures to better display the emphasis and meet the standards of journals and/or conferences. Furthermore, previous work has attempted to simplify the visualization process, e.g. PyMOL’s built-in “Publication” option provides a specified presentation scheme with a single button click, and the EZ-Viz plugin (Grell *et al.* 2006) transforms PyMOL’s multiple command sets into different tabs and buttons in order to simplify command interactions and user learning costs. However, the functions have not yet been effectively implemented. For example, the batch structure visualization function, the figure layout strategy that meets journal publishing standards, and the drawing process modules that are easier to use and understand.

To address these issues, we developed an open-source tool based on the PyMOL application (Schrödinger, LLC), named PyMOL-PUB. This tool provides integrated customizations for rapidly and efficiently constructing publication-quality figures of molecular structures. The optional functions in this tool include alignment methods, functional coloring schemes, conformation adjustments, and layout plotting strategies. As the tool follows the low coupling principle of design, users can combine these options with high flexibility. Meanwhile, it provides an easy-to-operate graphical user interface (GUI), demonstrations of single-step commands, and other comparable code examples that imitate figures from well-accepted papers. These efforts make it convenient for users to easily construct publication-quality figures through PyMOL-PUB.

## 2 Overview of PyMOL-PUB

PyMOL-PUB provides a unified plotting process for target figures. Typically, a figure to be built consists of two necessary layouts, statistical data and structure exhibition. The former can transmit plotting codes to the corresponding function through parameters. For the latter, a general structure exhibition process includes:

1. hide unimportant elements;
2. adjust structure states by translation, rotation, and scaling;
3. select specific parts of one or more structures;
4. specify the presentation mode and coloring scheme;
5. generate the high-resolution image.

For the structure exhibition process, some common functions are served to achieve different plotting effects:

- *Structure alignment:* Various widely-accepted structure alignment methods are integrated for numerical calculations of structural state transitions, including root-mean-square deviation (Petitjean 1999), global distance test (Zemla 2003), and template modeling score (Zhang and Skolnick 2004). We provide prerequisites before structure alignment, based on the applicability of different methods. With multi-dimensional matrix calculation, molecular structure alignments can be parallel processed in batches. These constrained methods provide reasonable observation angles and distances for each compared structure, minimizing visual differences between these structures.
- *Element selection:* Multi-level atomic selection commands are usually complex and require repeated empirical adjustments. An extensible and readable string expression is established as “*type: target, target,…,target*,” to configure elements at different levels. Here, “*type*” includes “atom,” “residue,” “position,” “range,” “segment,” “chain,” and “model.” With these simple phrases, users can directly specify the target element.
- *Coloring scheme:* PyMOL-PUB provides two optional coloring schemes, highlighting the selected elements, or coloring with gradients based on the attributes’ values. Such values can be the physical and chemical properties of a single object (deoxyribonucleic acids, ribonucleic acids, and amino acids) or the Euclidean distance between two objects with the same structure after comparison. Additionally, if the representation mode of the structure is “cartoon,” the volume size of the objects can also be adjusted to further emphasize numerical differences. The highly flexible coloring strategies can perform direct and visual demonstrations in the publications, such as molecular interaction, structure alignment, and structure prediction.

Furthermore, PyMOL-PUB supports the exhibition of multiple structural information via batch processing. Therefore, a series of high-resolution structure images can be pasted into the corresponding panels in the target figure. By building the entire process through code calls, users can obtain figures that meet their expectations. Thus, from the perspective of plotting targets, PyMOL-PUB can provide high-quality output for molecular interactions and structural comparison with batch processing.

**Figure 1.**
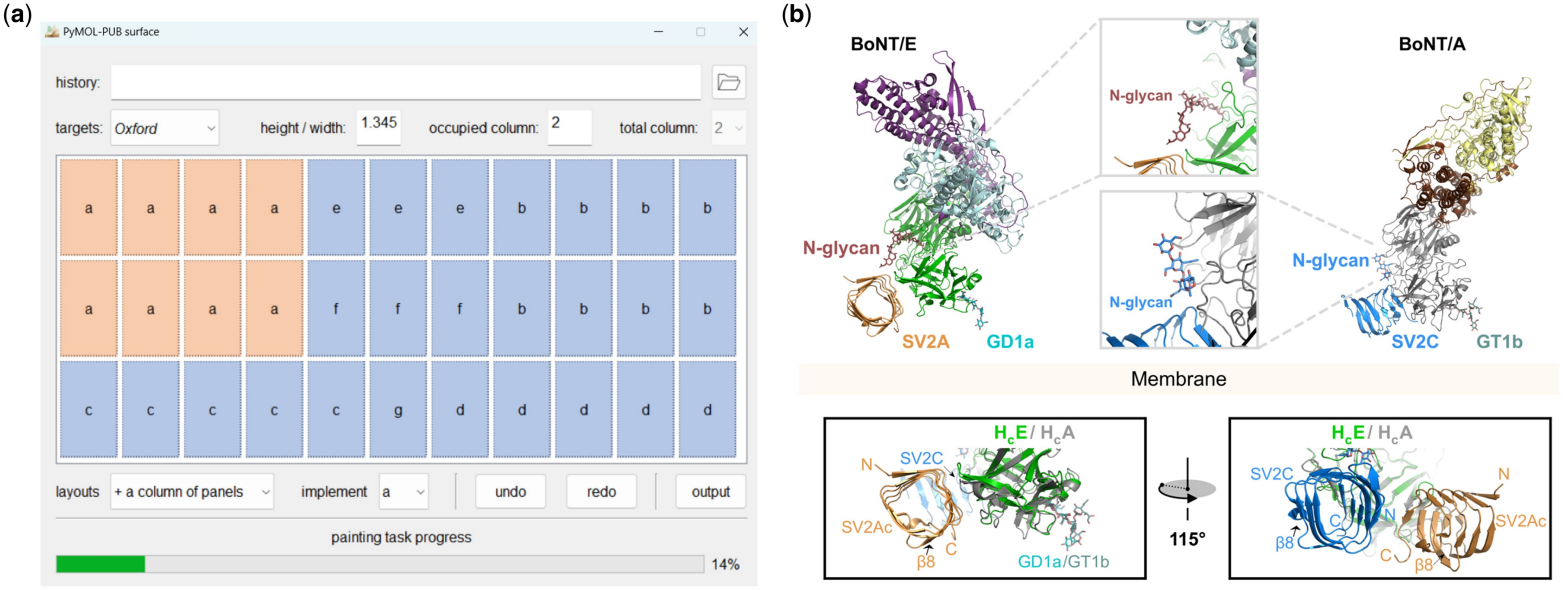
(a) Schematic representation of the main window of the GUI for PyMOL-PUB. The layout corresponds to the figure of panel (b). (b) A typical figure constructed by PyMOL-PUB with 116 lines of code. The original structure data and the basic figure layout refer to the published article (Liu *et al.* 2023), which illustrates most of the features of PyMOL-PUB, including structure comparison, batch operations, customized coloring, etc

To meet the plotting needs of users with different backgrounds, we provide two usage methods in PyMOL-PUB. For users who want to interact with the drawing process, PyMOL-PUB’s GUI can be used to create figures (Fig. 1a). Users can experience convenient and fast image parameter input and layout setting strategies. Through the general structure exhibition process described above, it is easy to customize the structure visualization method and place the drawing results in the designated panel. For deep users such as Python programmers, they can directly create target figures with a few lines of code (Fig. 1b), and experience various functions more flexibly, and then customize the figures. This is due to the fact that PyMOL-PUB allows input of the publication requirements during the figure initialization process, which eliminates the need for users to perform additional processing and post-processing on the figure after it is created. To sum up, users can flexibly combine and use various interfaces in PyMOL-PUB to efficiently customize and construct high-resolution figures for visualization, promoting the rapid development of structural biology.

## 3 Implementation

PyMOL-PUB is an open-source biomolecule structure visualization tool, which is programmed in Python 3 with PyMOL version 2.5. It can construct target structure images by calling several functions in Python application programming interfaces in PyMOL, such as “load,” “show,” “rotate,” “color,” and “png,” and integrate them into a complete figure for publication. Based on PyQt5 (Meier 2019), it can offer a process-oriented plotting GUI to replace the cost of programming Python scripts. Functions and interfaces of PyMOL-PUB have been successfully tested by CircleCI (Belmont 2018). Installation, configuration, customization, and other details can be found in its technical manual (Supplementary data).

## 4 Conclusion

PyMOL-PUB can assist users in self-designing bio-molecular structure figures conveniently, with less time and effort, to meet publication standards. Based on a given script with limited lines of code, users can produce figures repeatedly, without having to memorize the complicated plotting operations or steps as the conventional process. Alternatively, the process-oriented plotting GUI in PyMOL-PUB can guide users to construct complex figures through a process similar to software installation. These efforts make it convenient for users with different backgrounds to easily construct publication-quality figures.

Notably, these aforementioned scripts and their corresponding publication-quality figures also present unforeseen added value. These high-quality inputs and outputs can be widely disseminated like the gallery on the official website of *matplotlib* (Hunter 2007), helping to further reduce the learning curve for beginners. Hence, PyMOL-PUB not only aligns with the escalating voice of reproducibility but also advances community progression.

## Supplementary Material

btae139_Supplementary_Data

## Data Availability

No new data were generated or analysed in support of this research.

## References

[btae139-B1] Belmont J-M. Hands-On Continuous Integration and Delivery: Build and Release Quality Software at Scale with Jenkins, Travis CI, and CircleCI. United Kingdom: Packt Publishing Ltd, 2018.

[btae139-B2] DeLano WL. Pymol: an open-source molecular graphics tool. CCP4 Newsletter on Protein Crystallography 2002;40:82–92.

[btae139-B3] Grell L , Parkin C, Slatest L et al Ez-viz, a tool for simplifying molecular viewing in pymol. Biochem Mol Biol Educ 2006;34:402–7. 21638731 10.1002/bmb.2006.494034062672

[btae139-B4] Humphrey W , Dalke A, Schulten K. Vmd: visual molecular dynamics. J Mol Graph 1996;14:33–8. 8744570 10.1016/0263-7855(96)00018-5

[btae139-B5] Hunter JD. Matplotlib: a 2d graphics environment. Comput Sci Eng 2007;9:90–5.

[btae139-B6] Liu Z , Lee P-G, Krez N et al Structural basis for botulinum neurotoxin e recognition of synaptic vesicle protein 2. Nat Commun 2023;14:2338. 37095076 10.1038/s41467-023-37860-8 PMC10125960

[btae139-B7] Maveyraud L , Mourey L. Protein x-ray crystallography and drug discovery. Molecules 2020;25:1030. 32106588 10.3390/molecules25051030 PMC7179213

[btae139-B8] Meier B. Python GUI Programming Cookbook: Develop Functional and Responsive User Interfaces with tkinter and PyQt5. United Kingdom: Packt Publishing Ltd, 2019.

[btae139-B9] Petitjean M. On the root mean square quantitative chirality and quantitative symmetry measures. Journal of Mathematical Physics 1999;40:4587–95.

[btae139-B10] Pettersen EF , Goddard TD, Huang CC et al Ucsf chimera—a visualization system for exploratory research and analysis. J Comput Chem 2004;25:1605–12. 15264254 10.1002/jcc.20084

[btae139-B11] Sekhar A , Kay LE. An nmr view of protein dynamics in health and disease. Annu Rev Biophys 2019;48:297–319. 30901260 10.1146/annurev-biophys-052118-115647

[btae139-B12] Yip KM , Fischer N, Paknia E et al Atomic-resolution protein structure determination by cryo-em. Nature 2020;587:157–61. 33087927 10.1038/s41586-020-2833-4

[btae139-B13] Zemla A. Lga: a method for finding 3d similarities in protein structures. Nucleic Acids Res 2003;31:3370–4. 12824330 10.1093/nar/gkg571 PMC168977

[btae139-B14] Zhang Y , Skolnick J. Scoring function for automated assessment of protein structure template quality. Proteins: Structure, Function, and Bioinformatics 2004;57:702–10. 10.1002/prot.20264 15476259

