# Correction to: Rapid generation of high-quality structure figures for publication with PyMOL-PUB

**DOI:** 10.1093/bioinformatics/btae277

**Published:** 2024-04-27

This is a correction to: Yuting Chen, Haoling Zhang, Wen Wang, Yue Shen, Zhi Ping, Rapid generation of high-quality structure figures for publication with PyMOL-PUB, *Bioinformatics*, Volume 40, Issue 3, March 2024, btae139, https://doi.org/10.1093/bioinformatics/btae139.

In the originally published version of this manuscript, there was a typographic error in the first grant number given in the funding statement, which appeared as ‘2021YFF120010’ instead of ‘2021YFF1200100’.

This error has been corrected.

