# Supplementary material for: Rapid generation of high-quality structure figures for publication with PyMOL-PUB
Source: Bioinformatics. 2024 Mar 6;40(3):btae139. doi: 10.1093/bioinformatics/btae139 (PMC10950480; doi:10.1093/bioinformatics/btae139)
Supplement: btae139_Supplementary_Data [file btae139_supplementary_data.zip › PyMOL-PUB Technical Manual (clean).pdf]

PyMOL-PUB

Technical Manual

GitHub: https://github.com/BGI-SynBio/PyMOL-PUB

Version: v1.1

For more information, please contact:
zhanghaoling@genomics.cn and chenyuting@genomics.cn

Contents

1 Introduction **1**

2 Requirement analysis **1**
2.1 Function investigation 1
2.2 Function summary 2

3 Overview of PyMOL-PUB **3**

4 Installation **4**

5 Customization **5**
5.1 Publication-standard figure creation strategy 5
5.2 Structure image creation strategy 5
5.3 Structure attribute option 7
5.4 Structure selection strategy 7
5.5 Widget icon option 7

6 Single-step code line **9**
6.1 How to initialize figures based on publisher's requirements 9
6.2 How to create a structural image 10
6.3 How to set properties for the structure 12
6.4 How to select the parts of the structure(s) 13
6.5 How to obtain the specific rotation icon 14

7 Case study **15**
7.1 Case 1: protein-protein interaction 15
7.2 Case 2: protein structure alignment 17
7.3 Case 3: protein structure alignment and coloring by properties . . . 19
7.4 Case 4: structures batch visualization 21
7.5 Case 5: integrated usage 24

8 Usage of PyMOL-PUB surface (GUI) **27**
8.1 Interface logic 27
8.2 Main window 28
8.3 Selection window 29
8.4 Structure window 29
8.5 Statistics window 34

9 References **36**

List of Code Listings

1 Installation method based on “pip”. 4
2 Installation method after installing “git”. 5
3 Single-step of figure initialization. 10
4 Single-step of property visualization. 12
5 Single-step of basic part selection. 13
6 Single-step of accurate part selection. 13
7 Single-step of multiple part selection. 13
8 Single-step of the usage of part selection. 14
9 Single-step of rotation icon creations. 14
10 Plotting code lines of case 1. 16
11 Plotting code lines of case 2. 18
12 Plotting code lines of case 3. 20
13 Plotting code lines of case 4. 23
14 Plotting code lines of case 5 (part 1). 25
15 Plotting code lines of case 5 (part 2). 26

1 Introduction

With the rapid progress in fields such as protein structure prediction, an increasing number of researchers from different backgrounds require the use of PyMOL [1] for molecular visualization. To be used in publications, the default visualization output of PyMOL typically requires spatial adjustments, such as rotating and/or zooming the structures, and purposeful emphasis including highlighting important parts and hiding the unimportant parts. Meanwhile, the need for batch visualization has been demonstrated by recent publications in high-impact journals. However, these adjustments and batch protocols require the involvement of many skilled personnel, are expensive, and operate at human speeds, all of which make them worthy of automation. Based on the original design of PyMOL and as an important supplement, we have developed PyMOL-PUB to generate figures capable of reaching the publication standard. By using this tool, manual operations can be greatly reduced, and the desired figure output can be obtained with a few lines of code or operations in the Graphical User Interface (GUI).

2 Requirement analysis

2.1 Function investigation

To effectively establish PyMOL-PUB, we analyzed the molecular structure figures in recent publications [2, 3, 4, 5] from high-quality journals to determine the functions required by potential users. The illustration of required functions is shown in Figure 1. Therefore, PyMOL-PUB requires at least an alignment function, a batch layout function, and a highlighting (or even functional coloring) function to create publication-standard figures in a simpler and more rapid way.

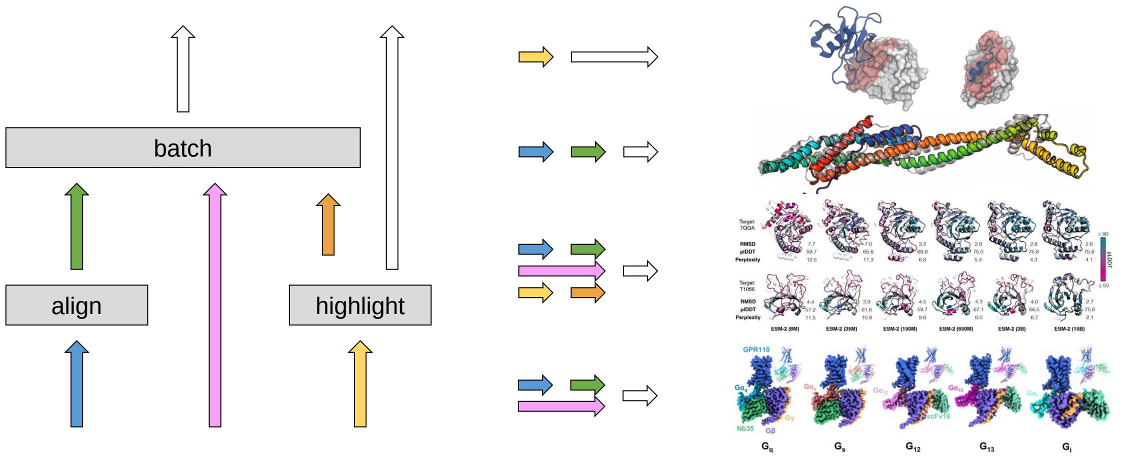

Figure 1: Functional analysis of well-established figures in recent publications

2.2 Function summary

Based on the clues from well-established publications provided in Figure 1, the functional modules are summarized as follows.

- **Alignment function**: In the process of structure alignment, structure rotation methods [6], scoring methods [7, 8, 9] and similarity metrics [10] are highly related to users' requirements. Furthermore, users may also have pre- and post-processing requirements like aligning parts of the structure or clustering the aligned structures [11, 9]. PyMOL-PUB considers the inclusion of the above-mentioned methods to further reduce the user's development costs and to have a positive impact on the user experience.

- **Batch layout function**: In general, the figure content in a high-quality publication tends to be extremely rich. For figures that need to show molecular structure information, a combination of multiple molecular structures occupies part of the figure or a panel [12]. A reasonable layout function for multiple molecules combined with the alignment function enables our tool to rapidly generate publication-quality figures.

- **Functional coloring function**: This function can regulate the coloring rule of the molecular structure to highlight the core information that users want to emphasize. Basic emphasis requirements include amino acids (or nucleotides), short segments, secondary structures and functional domains from micro to macro perspectives [12, 13, 14, 15]. Taking amino acids as a type of unit, a broader requirement is the coloring of each unit according to the provided value one by one. These values could be physicochemical properties of the units [16] or computational results of structure alignment [13]. Obvious properties include polarity, electronegativity, conformational flexibility, etc. Meanwhile, a classic case in alignment results is the root-mean-square deviation of each unit [7]. When these results are displayed for each unit in the predicted structure, the output figure can effectively show which parts of a particular structure are worse predicted by trained models.

- **Supporting functions**: Some functions are considered to improve the integrity of PyMOL-PUB. The general pre-processing functions are considered as a bridge between the modules mentioned above, which include loading structures from files, saving structures to files, decomposing the whole chain into sub-segments of a given k-mer length [17] and setting physicochemical properties for the chains. In order to generate publication-quality figures using the well-established plotting library *Matplotlib* [18], structure data and coloring data could be converted using the provided interfaces. Furthermore, format requirements (e.g. font, figure size, dots per inch, width limitation, etc.) of different journals and conferences are set when users utilize PyMOL-PUB to initialize the figures.

3 Overview of PyMOL-PUB

PyMOL-PUB exists to assist users in producing publication-quality figures in a rapid, efficient, and reusable way. The straightforward interaction between users and PyMOL-PUB is shown in Figure 2. Given the variety of users, we first concentrate on a specific group of users, i.e. programmers with experience in Python. PyMOL-PUB guides them in creating structural images. By sending plotting codes into its particular interface, it also enables users to present statistical data. The procedure is completed by integrating these two forms of plotting data into the target figure, and PyMOL-PUB is in charge of making sure the finished graph complies with the publication standards after programmers specify the target journal or conference. To enable a broader spectrum of users, notably those without Python programming experience, PyMOL-PUB provides a process-based plotting GUI. This group of users has the ability to create publication-quality figures through a visualization process similar to software installation. After multiple clicks and text inputs, the visualized operations could be transformed into a Python script, completing the painting of the desired figure as the final output.

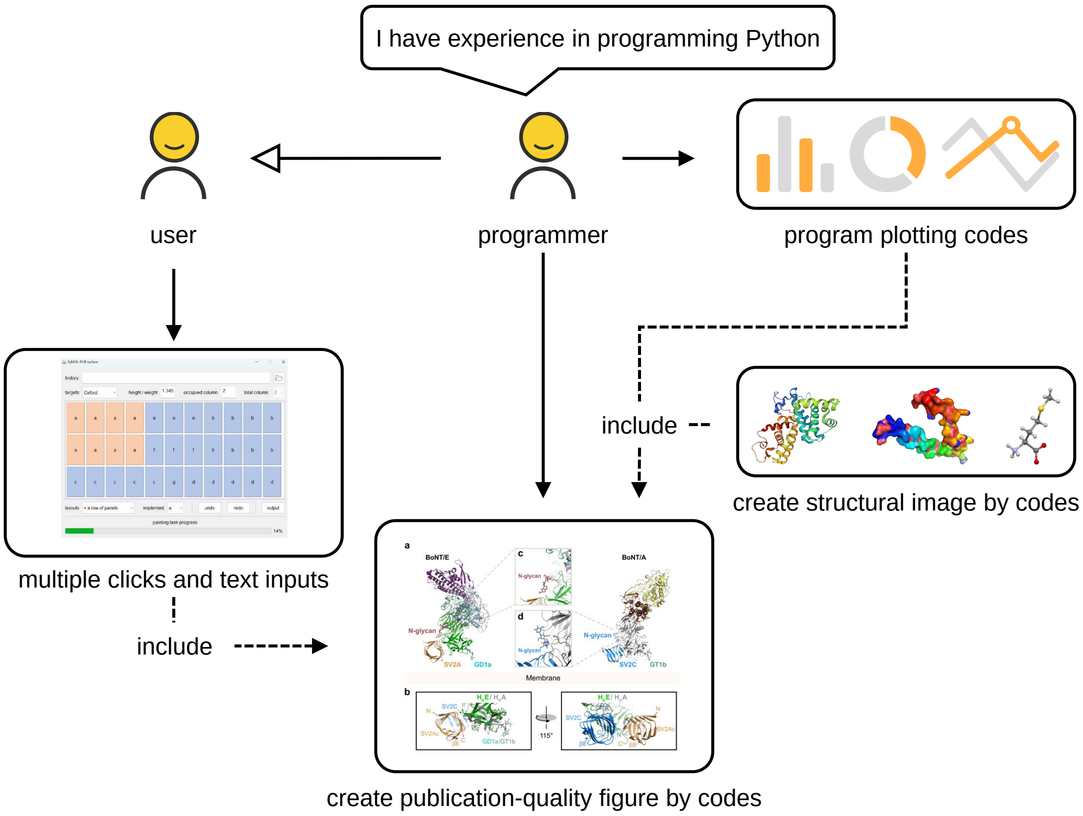

Figure 2: Case diagram.

The core code is composed of three Python scripts: “windows.py”, “handles.py”, and“layouts.py”. Among them, “windows.py” provides the source code for PyMOL-PUB GUI, running it can complete the overall plotting process; “handles.py” provides

a comprehensive set of operations for working with structured data, performing loading and saving operations, splicing and property assignment operations, and alignment-related calculations; “layouts.py” is responsible for figure processing and rendering operations. The file structure of the latter two scripts is shown in Figure 3.

handles

—[CLS] Monitor	The monitor used to identify the task progress.
—[CLS] Score	The similarity score between two molecule structures.
—[FUN] similar	Judge whether two structures are similar.
—[FUN] cluster	Cluster the molecule structures based on the similarity metric.
—[FUN] align	Align multiple molecule structures.
—[FUN] set_properties	Set physicochemical properties for structures.
—[FUN] set_difference	Set difference information for a known chain.
—[FUN] kmer	Disassemble the entire chain into sub segments of the given length.
—[FUN] load_structure_from_file	Load structure data from file.
—[FUN] save_structure_to_file	Save temporary structure to file.

layouts

—[CLS] Figure	Layout for displaying all information (including structures and data statistics).
—[CLS] DefaultStructureImage	Default structure image creator, supporting caching, zooming and translating functions.
—[CLS] HighlightStructureImage	Structure image creator for highlight predetermined parts of the structure.
—[CLS] PropertyStructureImage	Structure image creator for highlight the structure based on its element properties.
—[FUN] obtain_widget_icon	Obtain the widget icon (image) based on the predetermined setting.

CLS: class FUN: function

Figure 3: Repository structure.

4 Installation

Users may install it using pip directly (see Listing 1):

```
pip install PyMOL-PUB
```

Listing 1: Installation method based on “pip”.

Or users may install it from GitHub after installing git (see Listing 2):

```
git clone https://github.com/BGI-SynBio/PyMOL-PUB.git
cd PyMOL-PUB
pip install -r requirements.txt
python setup.py install develop --user
```

Listing 2: Installation method after installing "git".

The tool requires Python $\geq$ 3.7.3. As the foundation of this tool, we require a PyMOL version equal to 2.5.0. Please refer to the official website for installation protocol details. Additionally, some well-established libraries are required: Biopython $\geq$ 1.78, Matplotlib $\geq$ 3.2.0, Pillow $\geq$ 8.2.0, NumPy $\geq$ 1.21.2, SciPy $\geq$ 1.4.1, sphinx-rtd-theme $\geq$ 0.4.3 and PyQt5 $\geq$ 5.15.9 (a special dependency required by the GUI).

5 Customization

In this section, we describe 5 dimensions of figure customization. Corresponding single-step code lines and integrated case studies in Section 6 and 7, respectively.

5.1 Publication-standard figure creation strategy

PyMOL-PUB can customize the target publication format during the figure initialization. The supporting figure formats of journals, conferences, or publishers are shown in Table 1. Among them, the mathematical font is set as "*Linux Libertine* and *Lucida Calligraphy*" in this tool. For most journals in *Cell Press* [1], the journal-specific figure sizes contain 2-column and 3-column formats. PyMOL-PUB provides a parameter to specify the maximum number of columns (or total columns).

Once the figure has been initialized, it is possible to insert the generated structure image(s) into it by utilizing the "set_image" function or paint various outcomes, like line chart, bar chart, violin chart, and so on, through the "set_panel" function. The Figure class also provides a grid selection function since publications usually have varying information (i.e. aforementioned charts) to display in one figure.

5.2 Structure image creation strategy

For the structure image, two types of rendering objectives have been offered: the first aims to accentuate specific region(s), and the second intends to showcase element (deoxyribonucleic acid, ribonucleic acid, and amino acid) property information in the structure. In PyMOL-PUB, the HighlightStructureImage class can

[1] *AJHG*, *Cell*, *Cancer Cell*, *Cell Chemical Biology*, *Cell Genomics*, *Cell Host & Microbe*, *Cell Metabolism*, *Cell Reports*, *Cell Reports Medicine*, *Cell Stem Cell*, *Cell Systems*, *Current Biology*, *Developmental Cell*, *HGG Advances*, *Immunity*, *Molecular Cell*, *Molecular Therapy and sister journals*, *Neuron*, *One Earth*, *Patterns*, *Stem Cell Reports*, *Structure*, and *The Innovation*.

Target	Font	DPI	Total columns	Column-based width (inches)		
				1	2	3
Nature	*Arial*	300	2	3.54	7.08	-
Science	*Helvetica*	300	3	2.24	4.76	7.24
Cell	*Arial*	300	2	3.35	6.85	N.A.
			3	2.17	4.49	6.85
PNAS	*Helvetica*	600	2	3.42	7.00	N.A.
ACS	*Arial*	600	2	3.25	7.00	N.A.
Oxford	*Arial*	350	2	3.39	7.00	N.A.
PLOS	*Arial*	300	1	5.20	N.A.	N.A.
IEEE	*Times New Roman*	350	2	3.50	7.25	N.A.

Table 1: Available publication options.

offer adequate services for the former, whereas the PropertyStructureImage class is developed for the latter.

These two classes inherit from the DefaultStructureImage class (see Figure 3). Irrespective of the structure visualization method, as applicable in all cases, the recommended function calling order (not mandatory) is to [1] omit unnecessary parts by "set_cache" function; [2] modify the structure or its parts representation by "set_shape" function; [3] adjust the structure's spatial orientation by "set_state" function; [4] complete coloring of the structure or its parts by highlight coloring or property-driven coloring, both called "set_color" function in the corresponding class; [5] save the image by "save" function. The step-by-step case is shown in Figure 4, created by Line 5 - 15 in Listing 10.

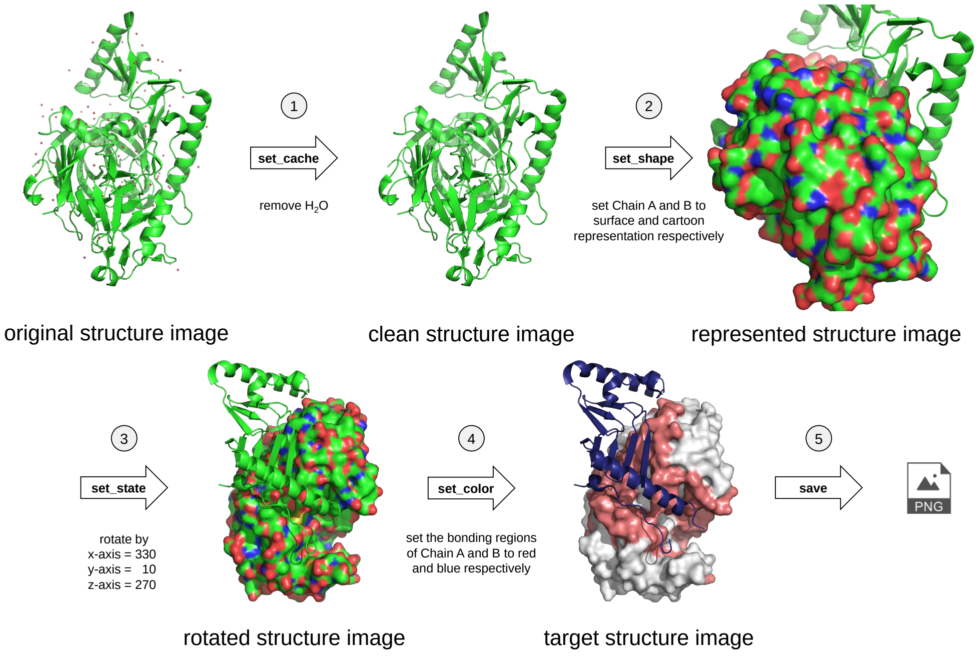

Figure 4: Step-by-step structure image creation.

5.3 Structure attribute option

More and more scientists realize that the molecular structure is highly related to its physicochemical properties [16]. Providing these properties can help users establish the relationship between biological functions and properties. Meanwhile, the association between properties and prediction results may help to further optimize trained models. Table 2 shows the collected properties for deoxyribonucleic acid ("DNA"), ribonucleic acid ("RNA"), and amino acids ("AA").

Element	Property	Description
DNA & RNA	ionization potentials	the amount of energy required to remove an electron from an isolated atom or molecule
DNA & RNA	electron affinities	the change in energy of a neutral atom when an electron is added to the atom to form a negative ion
AA	polarity	the distribution of electric charge
AA	turn propensity	the propensity of residues in turn structure
AA	helix propensity	the propensity of residues in alpha-helix structure
AA	sheet propensity	the propensity of residues in beta-sheet structure
AA	folding propensity	the propensity of residue be folded in protein structures, determined by hydrophobicity, secondary-structure propensity, and electrostatic charge
AA	flexibility	the conformational flexibility of residue, a crucial factor in determining their biological activity, including binding affinity, antigenicity, and enzymatic activity
all	electronegativity	the attraction a bonded atom has for electrons
all	hydrophobicity	the association of non-polar groups or molecules in an aqueous environment

Table 2: Available property options [19, 20, 21, 22, 23, 24, 25].

5.4 Structure selection strategy

We present a string expression to describe two selection types, one is "all" and another is "type:target,...,target", which avoids users needing to input extensive selection information based on the original PyMOL design. Here, "type" is a selection class, including "atom", "position", "range", "residue", "segment", "chain" and "model". "target" represents range(s) or object(s) under the corresponding "type".

5.5 Widget icon option

To better represent structural information, some icons are essential. Here, we provide two styles of rotation icons to describe the rotation of molecular structure. The widget is used to describe the rotation of the azimuth angle (x/y-axis) in style 1, which is shown in Figure 5.

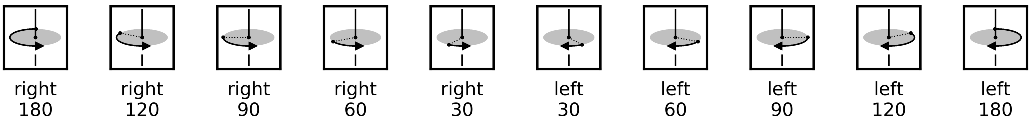

Figure 5: Rotation icon of style 1.

Style 2, as shown in Figure 6, further supports the elevation angle (z-axis).

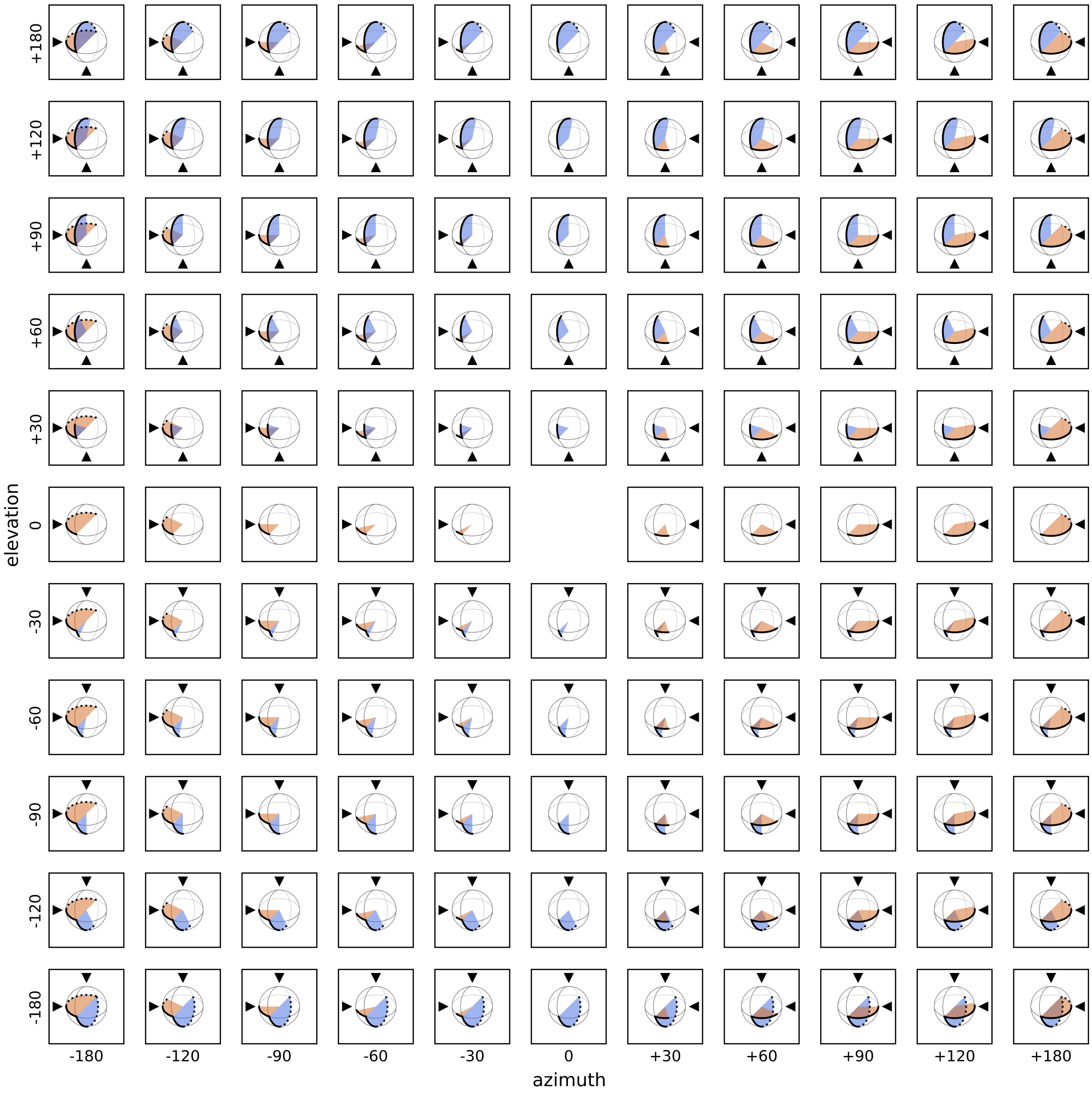

Figure 6: Rotation icon of style 2.

6 Single-step code line

This section serves as a derivation of the previous section in the implementation process. We provide a detailed description of the individual unit operations involved in achieving the painting objective. Users could refer to specific parts of this section to implement the code for a single step. For a more detailed interface interpretation, please refer to the GitHub repository and the ReadtheDocs website.

6.1 How to initialize figures based on publisher's requirements

The Figure object is instantiated with parameters that enable the achievement of the target publisher's requirements. The parameters in the initialization interface of the Figure class are as follows:

- **manuscript_format**: Format of the manuscript, including *Nature*, *Science*, *Cell*, *PNAS*, *ACS*, *Oxford*, *PLOS* and *IEEE*. When this parameter is passed in, the corresponding font style and minimum dots per inch (DPI) will also be automatically specified.

- **column_format**: Column format of the manuscript. As shown in Table 1, it only supports *Cell* format.

- **occupied_columns**: Occupied column number of the manuscript. It should not be greater than **column_format**. Based on **column_format** and **occupied_columns**, the width of the figure is determined.

- **aspect_ratio**: The aspect ratio of the figure (i.e. height:width). The input format is a tuple with the first value "height" and the last value "width". Due to the difficulty for users to set the figure height at the pixel value level, the intuitive aspect ratio helps users quickly achieve their expectations.

- **row_number** and **column_number**: Number of grid rows and columns in the figure. Due to the need for a single image to contain rich information, in most cases, structural images only occupy one panel or a part of the entire figure. Hence, users can use a grid layout to separate different information.

- **interval**: The horizontal (width) and vertical (height) space interval between panels, formed as a tuple.

Additionally, the parameter **dpi** allows users to improve figure clarity based on the minimum DPI proposed by the requirement.

Furthermore, some users may not be willing to use mathematical font styles, "*Linux Libertine* and *Lucida Calligraphy*", the parameter **mathtext** could be set as *False* (the default setting is *True*).

We provide 5 initialization examples of parameter combinations. Some referable codes and the corresponding annotations are shown in Listing 3. When the parameter combination is incorrect, the program will throw related exceptions.

```python
from molpub import Figure

# figure with Nature format (Arial font style and 300 dpi);
# the figure width is 7.08 inches (i.e. maximum width);
# height:width is 3:4;
# the entire figure has only one panel.
Figure(manuscript_format="Nature", occupied_columns=2, aspect_ratio=(3, 4))

# figure with Science format (Helvetica font style and 300 dpi);
# the figure width is 4.76 inches (i.e. two out of three columns);
# height:width is 1:2;
# the entire figure has two panels, on the left and right sides of the figure, respectively.
Figure(manuscript_format="Science", occupied_columns=2, aspect_ratio=(1, 2), row_number=1, column_number=2)

# figure with Cell format (Arial font style and 300 dpi);
# since 2-column format is used, the figure width is 6.85 inches (i.e. two out of two columns);
# height:width is 1:1;
# the entire figure has only one panel.
Figure(manuscript_format="Cell", column_format=2, occupied_columns=2, aspect_ratio=(1, 1))

# figure with Cell format (Arial font style and 300 dpi);
# since 3-column format is used, the figure width is 4.49 inches (i.e. two out of three columns);
# height:width is 1:1;
# the entire figure has only one panel.
Figure(manuscript_format="Cell", column_format=3, occupied_columns=2, aspect_ratio=(1, 1))

# figure with Oxford format (Arial font style and 350 dpi);
# the figure width is 7.00 inches (i.e. two out of two columns);
# height:width is 1:2;
# the entire figure has only one panel;
# the dpi has been elevated to 600.
Figure(manuscript_format="Oxford", occupied_columns=2, aspect_ratio=(1, 2), dpi=600)
```

Listing 3: Single-step of figure initialization.

6.2 How to create a structural image

Structural image creation involves 5 steps (omit unnecessary parts, modify the structure representation, adjust the structure's spatial orientation, color the structure, and save the image), a step-by-step case is shown in Figure 4. The parameter composition of the corresponding interface for each step is shown in Table 3.

Function	Interface	Parameter(s)
omit unnecessary parts	**set_cache**	**cache_contents**
modify the structure representation	**set_shape**	**representation_plan**, **initial_representation**, **independent_color**, **closed_surface**
adjust the structure's spatial orientation	**set_state**	**translate**, **rotate**, **inner_align**, **target**, **mobile**, **only_rotate**
	**set_zoom**	**zoom_contents**, **buffer**
color the structure	**set_color**	depends on the user's objectives (see Section 6.3 and 6.4)
save the image	**save**	**save_path**, **width**, **ratio**, **dpi**

Table 3: Interface description of structural image creation.

After creating an image object from the DefaultStructureImage class, the HighlightStructureImage class, or the PropertyStructureImage class, these functions can be directly used. The "close" interface should be accessed after saving the image to eliminate the impact of the current image creation on subsequent image creation. The brief parameter interpretation of the corresponding interfaces is as follows (detailed in the GitHub repository).

1. **set_cache**:
    - **cache_contents**: Hidden content list, e.g. water molecules.
2. **set_shape**:
    - **representation_plan**: The type of the visual structure.
    - **initial_representation**: Initial representation for all the structures, the default value is "cartoon". If the representation type is an index, can optionally operate on the specified chain.
    - **independent_color**: If this parameter is False, colors can leak into the open surface edge.
    - **closed_surface**: If this parameter is True, create a closed surface. Otherwise, the structural details inside could be visible.
3. **set_state**:
    - **translate**: Translate distances with x/y/z-axis.
    - **rotate**: Rotate degrees with x/y/z-axis.
    - **inner_align**: Align multiple structures through built-in interfaces (pymol2.PyMOL.align) if required.
    - **target**: The target (or template) name can be specified if **inner_align** is executed.
    - **mobile**: The mobile name can be specified if **inner_align** is executed.
    - **only_rotate**: Rotate without initialization.
4. **set_zoom**:
    - **zoom_contents**: Structure content that needs to be zoomed.
    - **buffer**: The buffer area size of the target structure.
5. **save**:
    - **save_path**: Path to save file.
    - **width**: Width of the structural image.
    - **ratio**: The ratio of width to height.
    - **dpi**: Dots per inch for the outputted image. The default setting is 1200, and we suggest that this parameter should not be less than 300, which is the minimum dpi required by most publishers.

Various examples (and related tricks) are represented in Section 7.

6.3 How to set properties for the structure

For the PropertyStructureImage class, the property data could be the similarity score (calculated by specific metrics; e.g. RMSD) or the physicochemical property (represented in Table 2) of the constituent elements of the displayed structure. Two examples are shown in Listing 4 using the root-mean-square deviation (RMSD) score and conformational flexibility [7, 19].

```
1  from molpub import set_properties, PropertyStructureImage
2  
3  file_parent_path = "./molecule/3/"
4  
5  # Alignment of the structure 7QQA and Predict, the visualization the Predict clearer.
6  properties = set_properties(structure_paths=[file_parent_path + "7QQA.pdb", file_parent_path + "Predict.pdb"],
7                              molecule_type="AA", property_type="PyMOL-align",
8                              targets=["chain:7QQA+A", "range:Predict+2-274"])
9  image_1 = PropertyStructureImage(structure_paths=[file_parent_path + "7QQA.pdb", file_parent_path + "Predict.pdb"])
10 image_1.set_cache(cache_contents=["model:7QQA"])
11 image_1.set_state(rotate=[330, 0, 0], inner_align=True, target="7QQA")
12 image_1.set_zoom(zoom_contents=["model:Predict"])
13 image_1.set_color(target="range:Predict+2-274", properties=properties, color_map="rainbow", gauge_strengthen=True)
14 image_1.save(save_path="a.png", width=1800, ratio=1.2)
15 image_1.close()
16 
17 # Description of the conformational flexibility of expected structure.
18 properties = set_properties(structure_paths=[file_parent_path + "7QQA.pdb"],
19                             molecule_type="AA", property_type="flexibility",
20                             targets=["chain:7QQA+A"])
21 image_2 = PropertyStructureImage(structure_paths=[file_parent_path + "7QQA.pdb", file_parent_path + "Predict.pdb"])
22 image_2.set_cache(cache_contents=["model:7QQA"])
23 image_2.set_state(rotate=[330, 0, 0], inner_align=True, target="7QQA")
24 image_2.set_zoom(zoom_contents=["model:Predict"])
25 image_2.set_color(target="range:Predict+2-274", properties=properties, color_map="rainbow", gauge_strengthen=True)
26 image_2.save(save_path="b.png", width=1800, ratio=1.2)
27 image_2.close()
```

Listing 4: Single-step of property visualization.

The code implemented in the property visualization is Line 6 – 8, 13, 18–20, 25. As a result, the initial action is to provide one or two structural details to receive matching property values. To establish the color, use the "set_color" interface and simply input the corresponding property values. Here, the corresponding images (a.png and b.png) of Listing 4 are shown in Figure 7.

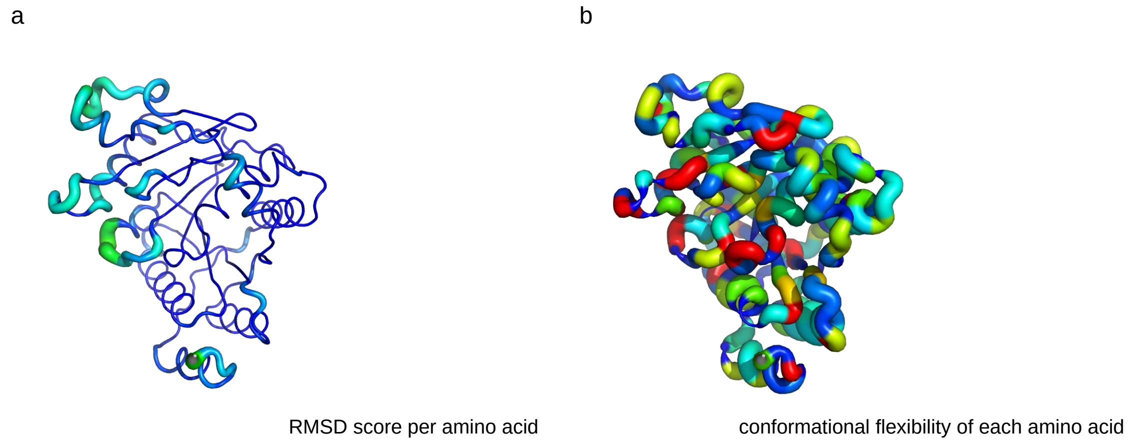

Figure 7: Structure property visualization based on the Listing 4.

6.4 How to select the parts of the structure(s)

Part selection strategy applies to the HighlightStructureImage class. To avoid users needing to input extensive selection information based on the original PyMOL design, we present a string expression to describe two selection types, one is "all" and another is "type:target,...,target". Here, "type" is the selection class, including (1) "atom", (2) "position", (3) "range", (4) "residue", (5) "segment", (6) "chain" and (7) "model". "target" represents the selection range under the corresponding "type".

Basically, users would like to select parts at different levels. Some string expressions are shown in Listing 5.

```
# select the model called "predicted".
a = "model:predicted"
# select A-chain.
b = "chain:A"
# select segment "NPGP" in all chains.
c = "segment:NPGP"
# select residue "HOH" in all chains.
d = "residue:HOH"
# select range from 10 to 20 and from 50 to 60 in all chains.
e = "range:10-20,50-60"
# select 10-th position in all chains.
f = "position:10"
```

Listing 5: Single-step of basic part selection.

For types below the chain scale, i.e. type (1) - (4), we provide a built-in chain description mechanism for more accurate selection (see Listing 6).

```
# select range from 10 to 20 in all chains.
a = "range:10-20"
# select range from 10 to 20 in A-chain.
b = "range:A+10-200"
# select the position 96 in A-chain.
c = "position:A+96"
```

Listing 6: Single-step of accurate part selection.

To make strings scalable, as shown in Listing 7, multiple targets can be declared within one string.

```
# select multiple residues including H2O, ADP, Mg, and SF4.
a = "residue:HOH,ADP,MG,SF4"
# select range 25~30, 50~55, 58~65, 70~73, 93~94, and 99~100 in A-chain.
b = "range:A+25-30,A+50-55,A+58-65,A+70-73,A+93-94,A+99-100"
```

Listing 7: Single-step of multiple part selection.

These string expressions are applied to omit("set_cache"), represent ("set_shape'), and color ("set_color") functions based on the parameter **cache_contents**, **representation_plan**, and **coloring_plan** (in the HighlightStructureImage class), respectively. Some referable codes are shown in Listing 8.

```python
from molpub import PropertyStructureImage, HighlightStructureImage

image_1 = PropertyStructureImage(structure_paths=["Predict.pdb"])
image_1.set_cache(cache_contents=["residue:HOH,ADP,MG,SF4"])

image_2 = HighlightStructureImage(structure_paths=["1F34.pdb"])
image_2.set_color(coloring_plan=[("chain:A", "0xF2F2F2"), ("chain:B", "0x2D2F82"),
                                 ("range:A+1-30,A+65-80,A+90-100,A+108-115,A+120-140", "0xF08080"),
                                 ("range:A+150-160,A+170-180,A+185-195,A+280-310", "0xF08080")])

image_3 = HighlightStructureImage(structure_paths=["1YCR.pdb"])
image_3.set_shape(representation_plan=[("chain:A", "surface"), ("chain:B", "cartoon")],
                  independent_color=True, closed_surface=True)
```

Listing 8: Single-step of the usage of part selection.

6.5 How to obtain the specific rotation icon

As mentioned in Section 5.5, we provide two styles of rotation icons. These rotation icons are created by the interface "obtain_widget_icon". Several program calls are represented in Listing 9.

```python
from molpub import obtain_widget_icon

# turn left 30 degrees.
obtain_widget_icon(save_path="a.png", widget_type="rotation", params={"turn": "right", "degree": 30})

# turn right 60 degrees.
obtain_widget_icon(save_path="b.png", widget_type="rotation", params={"turn": "left", "degree": 60})

# rotate according to elevation angle +30 degrees and azimuth angle +60 degrees.
obtain_widget_icon(save_path="c.png", widget_type="rotation", params={"elevation": +30, "azimuth": +60})

# rotate according to elevation angle -60 degrees and azimuth angle -30 degrees.
obtain_widget_icon(save_path="d.png", widget_type="rotation", params={"elevation": -60, "azimuth": -30})
```

Listing 9: Single-step of rotation icon creations.

The corresponding rotation icons are shown in Figure 8.

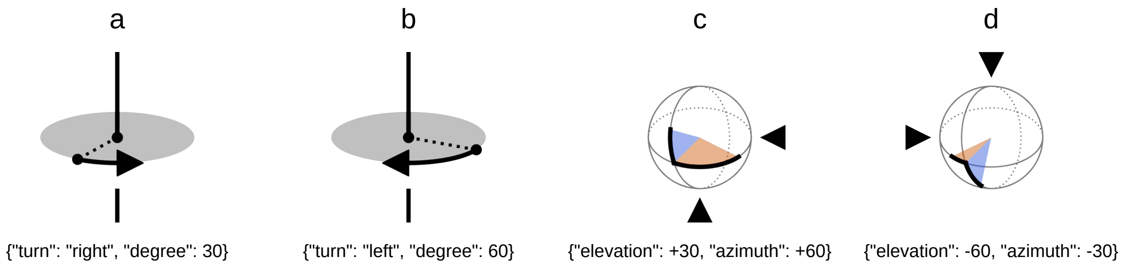

Figure 8: Rotation cases

This interface also supports creating a "line" or an "arrow" with the predetermined degree, referring to the source code for detailed parameter settings.

7 Case study

In this section, we present the rendered versions of the selected publications, highlighting the expected outcomes using PyMOL-PUB. Additionally, we offer some tricks employed in the painting process to facilitate constructive discussions towards similar representation objectives.

7.1 Case 1: protein-protein interaction

Figure 9 provides the repainting case of Corbi-Verge, et al. work [2], indicating that PyMOL-PUB supports the information representation of protein-protein interaction. Here, protein-protein interaction states are distinguished by setting specific representations and colors. Image annotations and widgets are also placed in the specified locations of the figure. For each complex, one of the interacting partners is displayed in blue cartoon representation, while the other is displayed in gray surface representation with the interface highlighted in red.

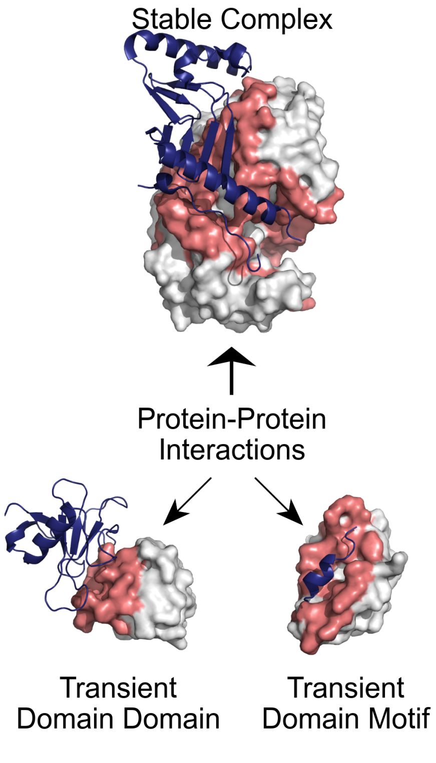

Figure 9: Protein–protein interaction

In the figure creation (see Listing 10), each structure was specified with a different representation based on chain A or B and a closed surface was created for the surface representation part using "closed_surface=True". The individual structure images are assigned a specific placement, as are the arrow widgets with specific angles.

```python
from molpub import DefaultStructureImage, HighlightStructureImage, Figure, obtain_widget_icon


def designed(file_parent_path, temp_parent_path, save_parent_path):
    # Visualization of the structure 1F34.
    s1f34 = HighlightStructureImage(structure_paths=[file_parent_path + "1F34.pdb"])
    s1f34.set_cache(cache_contents=["residue:HOH"])
    s1f34.set_shape(representation_plan=[("chain:A", "surface"), ("chain:B", "cartoon")],
                    independent_color=True, closed_surface=True)
    s1f34.set_state(rotate=[330, 10, 270])
    s1f34.set_color(coloring_plan=[("chain:A", "0xF2F2F2"), ("chain:B", "0x2D2F82"),
                                   ("range:A+1-30,A+65-80,A+90-100,A+108-115,A+120-140", "0xF08080"),
                                   ("range:A+150-160,A+170-180,A+185-195,A+280-310", "0xF08080")])
    s1f34.save(save_path=temp_parent_path + "1F34.png", width=1280, ratio=0.9)
    s1f34.close()

    # Visualization of the structure 1AY7.
    s1ay7 = HighlightStructureImage(structure_paths=[file_parent_path + "1AY7.pdb"])
    s1ay7.set_cache(cache_contents=["residue:HOH"])
    s1ay7.set_shape(representation_plan=[("chain:A", "cartoon"), ("chain:B", "surface")],
                    independent_color=True, closed_surface=True)
    s1ay7.set_state(rotate=[110, 30, 325])
    s1ay7.set_color(coloring_plan=[("chain:A", "0x2D2F82"), ("chain:B", "0xF2F2F2"), ("range:B+25-45", "0xF08080")])
    s1ay7.save(save_path=temp_parent_path + "1AY7.png", width=1280, ratio=1.2)
    s1ay7.close()

    # Visualization of the structure 1YCR.
    s1ycr = HighlightStructureImage(structure_paths=[file_parent_path + "1YCR.pdb"])
    s1ycr.set_shape(representation_plan=[("chain:A", "surface"), ("chain:B", "cartoon")],
                    independent_color=True, closed_surface=True)
    s1ycr.set_state(rotate=[250, 330, 255])
    s1ycr.set_color(coloring_plan=[("chain:A", "0xF2F2F2"), ("chain:B", "0x2D2F82"), ("position:A+96", "0xF08080"),
                                   ("range:A+25-30,A+50-55,A+58-65,A+70-73,A+93-94,A+99-100", "0xF08080")])
    s1ycr.save(save_path=temp_parent_path + "1YCR.png", width=1280, ratio=0.75)
    s1ycr.close()

    # Construct the case figure.
    case = Figure(manuscript_format="Nature", aspect_ratio=(606, 358))
    case.set_image(image_path=temp_parent_path + "1F34.png", layout=(1, 2, 1))
    case.set_image(image_path=temp_parent_path + "1AY7.png", layout=(2, 2, 3))
    case.set_image(image_path=temp_parent_path + "1YCR.png", layout=(2, 2, 4))
    case.set_text(annotation="Stable Complex", locations=[0.5, 0.96, 0.4, 0.05])
    case.set_text(annotation="Transient\nDomain Domain", locations=[0.25, 0.05, 0.4, 0.1])
    case.set_text(annotation="Transient\nDomain Motif", locations=[0.75, 0.05, 0.4, 0.1])
    case.set_text(annotation="Protein-Protein\nInteractions", locations=[0.5, 0.4, 0.4, 0.1])
    obtain_widget_icon(save_path=temp_parent_path + "arrow(90).png", widget_type="arrow", params={"degree": 90})
    case.set_image(image_path=temp_parent_path + "arrow(90).png", locations=[0.422, 0.44, 0.15, 0.15], transparent=True)
    obtain_widget_icon(save_path=temp_parent_path + "arrow(225).png", widget_type="arrow", params={"degree": 225})
    case.set_image(image_path=temp_parent_path + "arrow(225).png", locations=[0.325, 0.27, 0.15, 0.15], transparent=True)
    obtain_widget_icon(save_path=temp_parent_path + "arrow(315).png", widget_type="arrow", params={"degree": 315})
    case.set_image(image_path=temp_parent_path + "arrow(315).png", locations=[0.525, 0.27, 0.15, 0.15], transparent=True)
    case.save_figure(save_parent_path + "1.png")


if __name__ == "__main__":
    designed(file_parent_path="./molecule/1/", temp_parent_path="./temp/", save_parent_path="./designed/")
```

Listing 10: Plotting code lines of case 1.

7.2 Case 2: protein structure alignment

Figure 10 provides the repainting case of Yang, et al. work [3], indicating that PyMOL-PUB supports the information representation of protein structure alignment. Two structures are compared structurally. The expected structure and the predicted structure are shown in gray and rainbow cartoons, respectively.

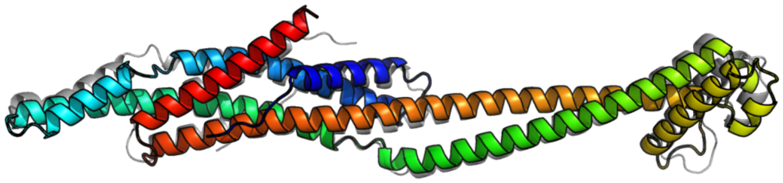

Figure 10: Protein structure alignment

In the figure creation (see Listing 11), adding a black border to a structure can be done by setting "edge_color" as black. It is worth noting that the border settings will be added for all structures in the view. So if you only want to add a border to one of the structures, you can only visualize the two structures separately and then overlay the images.

```python
from molpub import DefaultStructureImage, HighlightStructureImage, PropertyStructureImage, Figure


def designed(file_parent_path, temp_parent_path, save_parent_path):
    # Alignment of the structure T0950 and Predict, then visualization T0950.
    t0950 = HighlightStructureImage(structure_paths=[file_parent_path + "T0950.pdb", file_parent_path + "Predict.pdb"])
    t0950.set_cache(cache_contents=["model:Predict"])
    t0950.set_state(rotate=[120, 180, 0], inner_align=True, target="T0950")
    t0950.set_color(coloring_plan=[("model:T0950", "0xAAAAAA")])
    t0950.save(save_path=temp_parent_path + "T0950.png", width=1800, ratio=0.7)
    t0950.close()

    # Alignment of the structure T0950 and Predict, then visualization Predict.
    predict = PropertyStructureImage(structure_paths=[file_parent_path + "T0950.pdb",
                                                      file_parent_path + "Predict.pdb"])
    predict.set_cache(cache_contents=["model:T0950"])
    predict.set_state(rotate=[120, 180, 0], inner_align=True, target="T0950")
    predict.set_color(target="model:Predict", color_map="rainbow", edge_color="0x000000")
    predict.save(save_path=temp_parent_path + "Predict.png", width=1800, ratio=0.7)
    predict.close()

    # Construct the case figure.
    case = Figure(manuscript_format="Science", occupied_columns=2, aspect_ratio=(7, 10))
    case.set_image(image_path=temp_parent_path + "T0950.png", layout=(1, 1, 1))
    case.set_image(image_path=temp_parent_path + "Predict.png", layout=(1, 1, 1))
    case.save_figure(save_parent_path + "2.png")

    # Visualization and alignment of the structure T0950 and Predict(no outline).
    structure = PropertyStructureImage(structure_paths=[file_parent_path + "T0950.pdb",
                                                        file_parent_path + "Predict.pdb"])
    structure.set_state(rotate=[120, 180, 0], inner_align=True, target="T0950")
    structure.set_color(target="model:Predict", color_map="rainbow")
    structure.save(save_path=save_parent_path + "2(no outline).png", width=1800, ratio=0.7)
    structure.close()


if __name__ == "__main__":
    designed(file_parent_path="./molecule/2/", temp_parent_path="./temp/", save_parent_path="./designed/")
```

Listing 11: Plotting code lines of case 2.

7.3 Case 3: protein structure alignment and coloring by properties

Figure 11 provides the repainting case of Lin, et al. work [4], indicating that PyMOL-PUB supports the properties coloring of protein structure alignment. The visualization of conformational differences between expected and predicted structures is demonstrated. The predicted structure is shown in red-blue cartoons (colored by RMSD: red for low and blue for high) and the native structure is shown in white. Additionally, adjust the volume size of the predicted structure to further emphasize numerical differences shown in Figure 11b.

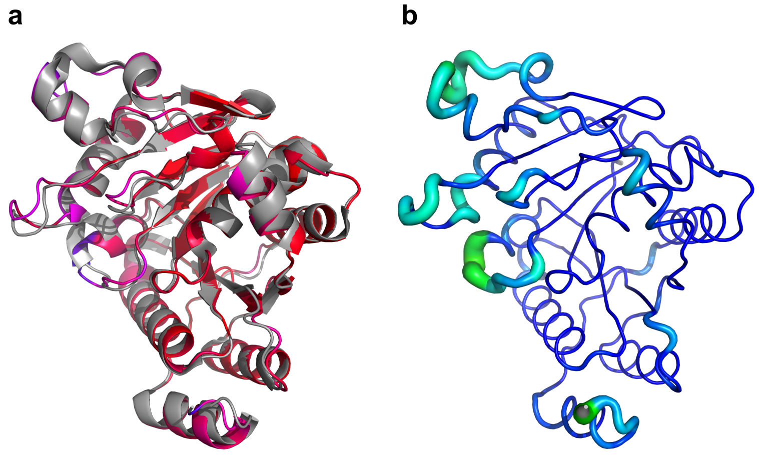

Figure 11: Protein structure alignment and coloring by properties

In the figure creation (see Listing 12), the first step is to calculate the physical or chemical properties of the selected structure using the "set_properties" function. Then, input the property values when designing the coloring scheme, so the structure is colored gradually according to the size of the value. Additionally, adjust the volume size of the predicted structure by setting "gauge_strengthen=True".

```python
from molpub import DefaultStructureImage, PropertyStructureImage, set_properties


def designed(file_parent_path, save_parent_path):
    # Visualization and alignment of the structure 7QQA and Predict.
    properties = set_properties(structure_paths=[file_parent_path + "7QQA.pdb", file_parent_path + "Predict.pdb"],
                                molecule_type="AA", property_type="PyMOL-align",
                                targets=["chain:7QQA+A", "range:Predict+2-274"])
    structure = PropertyStructureImage(structure_paths=[file_parent_path + "7QQA.pdb",
                                                        file_parent_path + "Predict.pdb"])
    structure.set_cache(cache_contents=["residue:HOH", "residue:ADP", "residue:MG", "residue:SF4",
                                        "chain:7QQA+B", "chain:7QQA+C", "chain:7QQA+D"])
    structure.set_state(rotate=[330, 0, 0], inner_align=True, target="7QQA")
    structure.set_zoom(zoom_contents=["model:Predict"])
    structure.set_color(target="range:Predict+2-274", properties=properties, color_map="rmbc")
    structure.save(save_path=save_parent_path + "3.png", width=1800, ratio=1.2)
    structure.close()

    # Alignment of the structure 7QQA and Predict, the visualization the Predict clearer.
    properties = set_properties(structure_paths=[file_parent_path + "7QQA.pdb", file_parent_path + "Predict.pdb"],
                                molecule_type="AA", property_type="PyMOL-align",
                                targets=["chain:7QQA+A", "range:Predict+2-274"])
    structure = PropertyStructureImage(structure_paths=[file_parent_path + "7QQA.pdb",
                                                        file_parent_path + "Predict.pdb"])
    structure.set_cache(cache_contents=["model:7QQA"])
    structure.set_state(rotate=[330, 0, 0], inner_align=True, target="7QQA")
    structure.set_zoom(zoom_contents=["model:Predict"])
    structure.set_color(target="range:Predict+2-274", properties=properties, color_map="rainbow", gauge_strengthen=True)
    structure.save(save_path=save_parent_path + "3(clearer).png", width=1800, ratio=1.2)
    structure.close()


if __name__ == "__main__":
    designed(file_parent_path="./molecule/3/", save_parent_path="./designed/")
```

Listing 12: Plotting code lines of case 3.

7.4 Case 4: structures batch visualization

Figure 12 provides the repainting case of Zhu, et al. work [5], indicating that PyMOL-PUB supports batch visualization of structures. The overall structures of GPR110/Gq, GPR110/Gs, GPR110/Gi, GPR110/G12 and GPR110/G13 complexes are displayed. The images of each structure and annotations are placed in order at the specified locations in the figure. Each subunit has its assigned color.

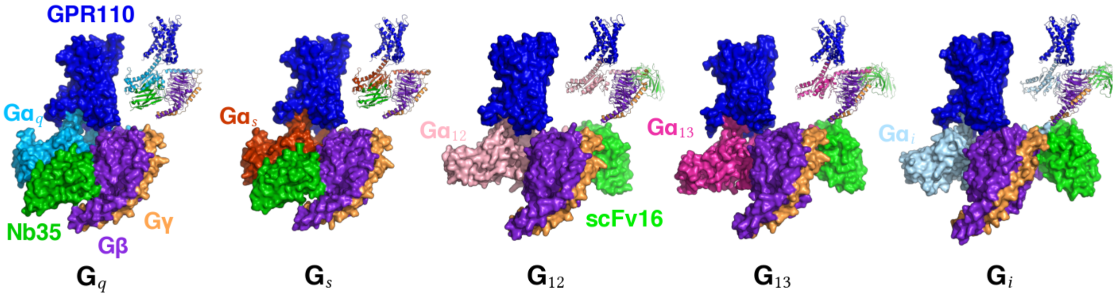

Figure 12: Structures batch visualization

In the figure creation (see Listing 13), the different chains of each complex are assigned a particular color. Divide the entire layer into five panels, and place each structure image in its location in the corresponding panel. The corresponding annotation text for each structure is also placed in the specified location.

```python
from molpub import DefaultStructureImage, HighlightStructureImage, Figure


def designed(file_parent_path, temp_parent_path, save_parent_path):
    # Visualization of the structure GPR110Gq.
    gpr110gq = HighlightStructureImage(structure_paths=[file_parent_path + "GPR110Gq.pdb"])
    gpr110gq.set_state(rotate=[180, 15, 90])
    gpr110gq.set_shape(representation_plan=[("all", "cartoon")])
    gpr110gq.set_color(coloring_plan=[("chain:A", "0x00BFFF"), ("chain:B", "0x8A2BE2"), ("chain:G", "0xFFA54F"),
                                      ("chain:N", "0x00CD00"), ("chain:R", "0x0000EE")])
    gpr110gq.save(save_path=temp_parent_path + "GPR110Gq-cartoon.png", width=1280, ratio=1.0)
    gpr110gq.set_shape(representation_plan=[("all", "surface")])
    gpr110gq.save(save_path=temp_parent_path + "GPR110Gq-surface.png", width=1280, ratio=1.0)
    gpr110gq.close()

    # Visualization of the structure GPR110Gs.
    gpr110gs = HighlightStructureImage(structure_paths=[file_parent_path + "GPR110Gs.pdb"])
    gpr110gs.set_state(rotate=[0, 15, 90])
    gpr110gs.set_shape(representation_plan=[("all", "cartoon")])
    gpr110gs.set_color(coloring_plan=[("chain:A", "0xCD3700"), ("chain:B", "0x8A2BE2"), ("chain:C", "0xFFA54F"),
                                      ("chain:D", "0x00CD00"), ("chain:R", "0x0000EE")])
    gpr110gs.save(save_path=temp_parent_path + "GPR110Gs-cartoon.png", width=1280, ratio=1.0)
    gpr110gs.set_shape(representation_plan=[("all", "surface")])
    gpr110gs.save(save_path=temp_parent_path + "GPR110Gs-surface.png", width=1280, ratio=1.0)
    gpr110gs.close()

    # Visualization of the structure GPR110G12.
    gpr110g12 = HighlightStructureImage(structure_paths=[file_parent_path + "GPR110G12.pdb"])
    gpr110g12.set_state(rotate=[30, 345, 135])
    gpr110g12.set_shape(representation_plan=[("all", "cartoon")])
    gpr110g12.set_color(coloring_plan=[("chain:A", "0xFFB5C5"), ("chain:B", "0x8A2BE2"), ("chain:C", "0x00EE00"),
                                       ("chain:E", "0xFFA54F"), ("chain:R", "0x0000EE")])
    gpr110g12.save(save_path=temp_parent_path + "GPR110G12-cartoon.png", width=1280, ratio=1.0)
    gpr110g12.set_shape(representation_plan=[("all", "surface")])
    gpr110g12.save(save_path=temp_parent_path + "GPR110G12-surface.png", width=1280, ratio=1.0)
    gpr110g12.close()

    # Visualization of the structure GPR110G13.
    gpr110g13 = HighlightStructureImage(structure_paths=[file_parent_path + "GPR110G13.pdb"])
    gpr110g13.set_state(rotate=[330, 0, 315])
    gpr110g13.set_shape(representation_plan=[("all", "cartoon")])
    gpr110g13.set_color(coloring_plan=[("chain:A", "0xEE30A7"), ("chain:B", "0x8A2BE2"), ("chain:C", "0x00EE00"),
                                       ("chain:D", "0xFFA54F"), ("chain:R", "0x0000EE")])
    gpr110g13.save(save_path=temp_parent_path + "GPR110G13-cartoon.png", width=1280, ratio=1.0)
    gpr110g13.set_shape(representation_plan=[("all", "surface")])
    gpr110g13.save(save_path=temp_parent_path + "GPR110G13-surface.png", width=1280, ratio=1.0)
    gpr110g13.close()

    # Visualization of the structure GPR110Gi.
    gpr110gi = HighlightStructureImage(structure_paths=[file_parent_path + "GPR110Gi.pdb"])
    gpr110gi.set_state(rotate=[165, 0, 315])
    gpr110gi.set_shape(representation_plan=[("all", "cartoon")])
    gpr110gi.set_color(coloring_plan=[("chain:B", "0xB0E2FF"), ("chain:C", "0x8A2BE2"), ("chain:E", "0x00EE00"),
                                      ("chain:D", "0xFFA54F"), ("chain:R", "0x0000EE")])
    gpr110gi.save(save_path=temp_parent_path + "GPR110Gi-cartoon.png", width=1280, ratio=1.0)
    gpr110gi.set_shape(representation_plan=[("all", "surface")])
    gpr110gi.save(save_path=temp_parent_path + "GPR110Gi-surface.png", width=1280, ratio=1.0)
    gpr110gi.close()

    # Construct the case figure.
    case = Figure(manuscript_format="Science", occupied_columns=2, aspect_ratio=(1.54, 5))
    for order, structure_name in enumerate(["GPR110Gq", "GPR110Gs", "GPR110G12", "GPR110G13", "GPR110Gi"]):
        case.set_image(image_path=temp_parent_path + structure_name + "-surface.png", layout=(5, 1, order + 1))
        case.set_image(image_path=temp_parent_path + structure_name + "-cartoon.png",
                       locations=[(0.11 + order * 0.2), 0.45, 0.11, 0.5], transparent=True)
        case.set_text(annotation="G$_{" + structure_name[7::] + "}$", locations=[(0.1 + order * 0.2), 0.1, 0.2, 0.2],
                      font_size=8, weight="bold", transparent=True)
    text_dict = {1: ("GPR110", [0.1, 0.85, 0.2, 0.2], "0000EE"), 2: ("Gα$_{q}$", [0.04, 0.57, 0.2, 0.2], "00BFFF"),
                 3: ("Nb35", [0.05, 0.23, 0.2, 0.2], "00CD00"), 4: ("Gβ", [0.12, 0.19, 0.2, 0.2], "8A2BE2"),
                 5: ("Gγ", [0.16, 0.26, 0.2, 0.2], "FFA54F"), 6: ("Gα$_{s}$", [0.23, 0.56, 0.2, 0.2], "CD3700"),
                 7: ("Gα$_{12}$", [0.41, 0.52, 0.2, 0.2], "FFB5C5"), 8: ("scFv16", [0.57, 0.27, 0.2, 0.2], "00EE00"),
                 9: ("Gα$_{13}$", [0.61, 0.53, 0.2, 0.2], "EE30A7"), 10: ("Gα$_{i}$", [0.81, 0.51, 0.2, 0.2], "B0E2FF")}
    for _, content in text_dict.items():
        case.set_text(annotation=content[0], locations=content[1], font_size=7, weight="bold", color="#" + content[2],
                      transparent=True)
    case.save_figure(save_parent_path + "4.png")


if __name__ == "__main__":
    designed(file_parent_path="./molecule/4/", temp_parent_path="./temp/", save_parent_path="./designed/")
```

Listing 13: Plotting code lines of case 4.

7.5 Case 5: integrated usage

The figure in the manuscript (also attached in Figure 13) provides the repainting case of Liu, et al. work [26], which shows an integration case. First are structural visualizations of two protein complexes, and each subunit has a specific color. Then, the two structures are superimposed based on the highly similar subunits, and differences in structural alignment can be distinguished by their different colors. Finally, the N-glycan ligand and protein interaction details can be explored in this figure. Additionally, the complete figure layout is based on simple batch operations.

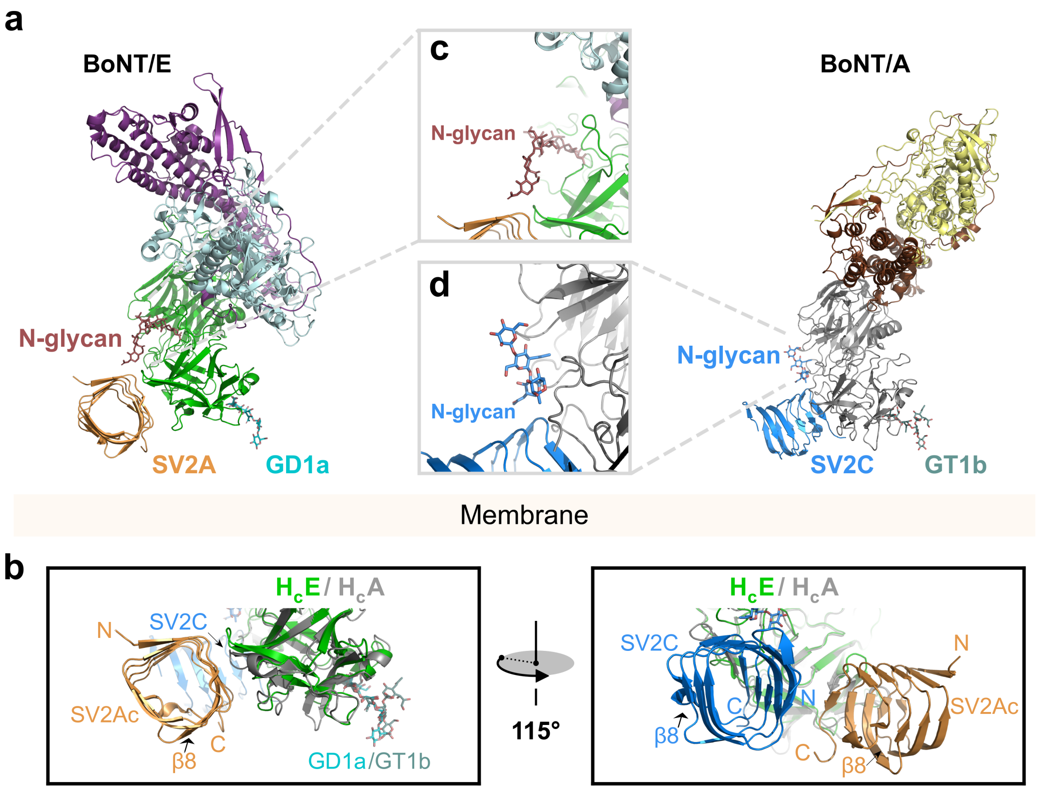

Figure 13: Integrated usage

In the structure image creation (see Listing 14), multiple structures are aligned based on highly similar subunits and all excess is hidden. Use the "set_zoom" interface to focus on the target section and visualize the specified details.

```python
from molpub import DefaultStructureImage, HighlightStructureImage, Figure, obtain_widget_icon


def designed(file_parent_path, temp_parent_path, save_parent_path):
    # Alignment of the structure 3FFZ, 3QUM, 7OVW and 7UIA, then visualization.
    complex_e = HighlightStructureImage(structure_paths=[file_parent_path + "3FFZ.pdb", file_parent_path + "3QUM.pdb",
                                                         file_parent_path + "7OVW.cif", file_parent_path + "7UIA.pdb"])
    complex_e.set_state(inner_align=True, target="(3FFZ and chain A)", mobile="(7UIA and chain A)")
    complex_e.set_state(inner_align=True, target="(3FFZ and chain A)", mobile="(7OVW and chain AAA)")
    complex_e.set_state(inner_align=True, target="(7UIA and chain G)", mobile="(3QUM and chain G)")
    complex_e.set_cache(cache_contents=["residue:HOH,ZN,NA,ACT,SO4,PEG,A2G", "chain:7OVW+AAA,7OVW+BBB",
                                        "residue:7UIA+G+NAG,7UIA+C+NAG,7UIA+H+NAG,7UIA+F+NAG", "range:3QUM+G+8-15",
                                        "residue:3QUM+E+NAG,3QUM+E+MAN,3QUM+E+GAL,3QUM+E+SIA,3QUM+E+FUC,3QUM+F+GAL",
                                        "residue:7OVW+HHH+BGC,7OVW+HHH+GAL,7OVW+HHH+NGA,7OVW+HHH+SIA", "chain:3FFZ+B",
                                        "chain:3QUM+A,3QUM+B,3QUM+C,3QUM+D,3QUM+H,3QUM+K,3QUM+L,3QUM+M,3QUM+P,3QUM+Q",
                                        "chain:7UIA+A,7UIA+B,7UIA+D,7UIA+E,7UIA+F,7UIA+H,7UIA+G", "atom:7OVW+GGG+H"])
    complex_e.set_color(coloring_plan=[("range:3FFZ+A+1-400", "0xCAFBFC"), ("range:3FFZ+A+401-800", "0x8C3E97"),
                                       ("range:3FFZ+A+801-1252", "0x00CD00"), ("chain:7UIA+C", "0xE59747"),
                                       ("chain:3QUM+G", "0xA15153"), ("chain:7OVW+GGG", "0x00C5CD"),
                                       ("atom:7OVW+GGG+O,7OVW+GGG+H", "0xC07079")])
    complex_e.set_state(rotate=[280, 5, 240])
    complex_e.set_zoom(zoom_contents=["chain:3FFZ+A"], buffer=5.0)
    complex_e.save(save_path=temp_parent_path + "BoNTE-SV2-gangliosides.png", width=1800, ratio=1.2)
    complex_e.set_zoom(zoom_contents=["chain:3QUM+G"], buffer=1.0)
    complex_e.save(save_path=temp_parent_path + "BoNTE-SV2-gangliosides-zoom(N-glycan).png", width=1800, ratio=1.0)
    complex_e.close()

    # Alignment of the structure 3BTA, 5JLV, 2VU9, then visualization.
    complex_a = HighlightStructureImage(structure_paths=[file_parent_path + "3BTA.pdb", file_parent_path + "5JLV.pdb",
                                                         file_parent_path + "2VU9.pdb"])
    complex_a.set_state(inner_align=True, target="(3BTA)", mobile="(5JLV and chain A)")
    complex_a.set_state(inner_align=True, target="(3BTA)", mobile="(2VU9)")
    complex_a.set_cache(cache_contents=["residue:HOH,MG,ZN,ACT,PO4", "residue:5JLV+F+NAG,5JLV+F+FUC,5JLV+C+NAG",
                                        "chain:5JLV+B,5JLV+A,5JLV+D", "chain:2VU9+A"])
    complex_a.set_color(coloring_plan=[("range:3BTA+A+1-450", "0xFCFC82"), ("range:3BTA+A+451-875", "0x884423"),
                                       ("range:3BTA+A+876-1295", "0x999999"), ("chain:5JLV+C", "0x0989FC"),
                                       ("chain:5JLV+E", "0x3892F3"), ("atom:5JLV+E+O,5JLV+E+H", "0xD55758"),
                                       ("chain:2VU9+B", "0x639592"), ("atom:2VU9+B+O,2VU9+B+H", "0xD55758")])
    complex_a.set_state(rotate=[65, 12.5, 50])
    complex_a.set_zoom(zoom_contents=["model:3BTA"], buffer=20.0)
    complex_a.save(save_path=temp_parent_path + "BoNTA-SV2-gangliosides.png", width=1800, ratio=1.2)
    complex_a.set_zoom(zoom_contents=["chain:5JLV+E"], buffer=10.0)
    complex_a.save(save_path=temp_parent_path + "BoNTA-SV2-gangliosides-zoom(N-glycan).png", width=1800, ratio=1.0)
    complex_a.close()

    # Alignment of the structure 7UIA, 5JLV, 7OVW and 2VU9, then visualization.
    complexsub = HighlightStructureImage(structure_paths=[file_parent_path + "7UIA.pdb", file_parent_path + "5JLV.pdb",
                                                          file_parent_path + "7OVW.cif", file_parent_path + "2VU9.pdb"])
    complexsub.set_state(inner_align=True, target="(7UIA and chain A)", mobile="(5JLV and chain A)")
    complexsub.set_state(inner_align=True, target="(7UIA and chain A)", mobile="(7OVW and chain AAA)")
    complexsub.set_state(inner_align=True, target="(5JLV and chain A)", mobile="(2VU9)")
    complexsub.set_cache(cache_contents=["residue:HOH,MG,ZN,NA,ACT,PO4,SO4,PEG,A2G", "chain:7OVW+AAA,7OVW+BBB",
                                         "residue:7UIA+G+NAG,7UIA+C+NAG,7UIA+H+NAG,7UIA+F+NAG", "chain:5JLV+B,5JLV+D",
                                         "residue:7OVW+HHH+BGC,7OVW+HHH+GAL,7OVW+HHH+NGA,7OVW+HHH+SIA", "chain:2VU9+A",
                                         "residue:5JLV+F+NAG,5JLV+F+FUC,5JLV+C+NAG", "atom:7OVW+GGG+H",
                                         "chain:7UIA+B,7UIA+D,7UIA+E,7UIA+F,7UIA+H,7UIA+G"])
    complexsub.set_color(coloring_plan=[("chain:7UIA+A", "0x00CD00"), ("chain:7UIA+C", "0xE59747"),
                                        ("chain:7OVW+GGG", "0x00C5CD"), ("atom:7OVW+GGG+O,7OVW+GGG+H", "0xC07079"),
                                        ("chain:5JLV+A", "0x999999"), ("chain:5JLV+C", "0x0989FC"),
                                        ("chain:5JLV+E", "0x3892F3"), ("atom:5JLV+E+O,5JLV+E+H", "0xD55758"),
                                        ("chain:2VU9+B", "0x639592"), ("atom:2VU9+B+O,2VU9+B+H", "0xD55758")])
    complexsub.set_state(rotate=[205, 20, 25])
    complexsub.set_zoom(zoom_contents=["range:7UIA+A+1102-1103"], buffer=18.0)
    complexsub.save(save_path=temp_parent_path + "HCE_SV2Ac-HCA_SV2C.png", width=1800, ratio=0.5)
    complexsub.set_state(rotate=[0, 115, 0], only_rotate=True)
    complexsub.set_zoom(zoom_contents=["range:7UIA+C+576-577"], buffer=18.0)
    complexsub.save(save_path=temp_parent_path + "HCE_SV2Ac-HCA_SV2C(115).png", width=1800, ratio=0.5)
    complexsub.close()


if __name__ == "__main__":
    designed(file_parent_path="./molecule/manuscript/", temp_parent_path="./temp/", save_parent_path="./designed/")
```

Listing 14: Plotting code lines of case 5 (part 1).

And in the figure creation (see Listing 15), multiple widgets such as “rotation”, “arrow”, and “line” are added to the figure. When setting images and texts, users can also set some customized parameters such as “border-color”, “border-size”, “font-size”, and “font-weight”. These integrated customized operations help users create target figures more easily and flexibly.

```python
from molpub import DefaultStructureImage, HighlightStructureImage, Figure, obtain_widget_icon


def designed(file_parent_path, temp_parent_path, save_parent_path):
    # Construct the case figure.
    case = Figure(manuscript_format="Oxford", occupied_columns=2, aspect_ratio=(1298, 1746), dpi=600, mathtext=True)
    # construct widgets
    widget_icon_dict = {"rotation(115)": {"turn": "right", "degree": 115}, "arrow(45)": {"degree": 45},
                        "arrow(315)": {"degree": 315}, "arrow(85)": {"degree": 85},
                        "line(45)": {"degree": 45, "linestyle": "--", "linewidth": 0.992, "color": "#D9D9D9"},
                        "line(25)": {"degree": 25, "linestyle": "--", "linewidth": 1.2, "color": "#D9D9D9"},
                        "line(155)": {"degree": 155, "linestyle": "--", "linewidth": 2.232, "color": "#D9D9D9"},
                        "line(30)": {"degree": 30, "linestyle": "--", "linewidth": 2.232, "color": "#D9D9D9"}}
    for wname, content in widget_icon_dict.items():
        obtain_widget_icon(save_path=temp_parent_path + wname + ".png", widget_type=wname.split('(')[0], params=content)
    # set structure images
    case.set_image(image_path=temp_parent_path + "BoNTE-SV2-gangliosides.png", locations=[0.075, 0.35, 0.3, 0.65])
    case.set_image(image_path=temp_parent_path + "BoNTA-SV2-gangliosides.png", locations=[0.675, 0.35, 0.3, 0.65])
    case.set_image(image_path=temp_parent_path + "HCE_SV2Ac-HCA_SV2C.png", locations=[0.08, 0.01, 0.38, 0.35],
                   positions=(8, 4, 0, 8, -0.5, 3.25), frame_off=False, linewidth=1.5)
    case.set_image(image_path=temp_parent_path + "HCE_SV2Ac-HCA_SV2C(115).png", locations=[0.56, 0.01, 0.38, 0.35],
                   positions=(8, 4, 0.5, 8.45, -0.5, 3.25), frame_off=False, linewidth=1.5)
    case.set_image(image_path=temp_parent_path + "BoNTE-SV2-gangliosides-zoom(N-glycan).png",
                   locations=[0.375, 0.7, 0.25, 0.25], frame_off=False, linewidth=1.5, linecolor="#D9D9D9")
    case.set_image(image_path=temp_parent_path + "BoNTA-SV2-gangliosides-zoom(N-glycan).png",
                   locations=[0.375, 0.425, 0.25, 0.25], frame_off=False, linewidth=1.5, linecolor="#D9D9D9")
    # set widgets
    widget_image_dict = {1: ("rotation(115)", [0.46, 0.15, 0.1, 0.1]), 2: ("arrow(315)", [0.215, 0.215, 0.025, 0.025]),
                         3: ("arrow(85)", [0.19, 0.09, 0.025, 0.025]), 4: ("arrow(85)", [0.625, 0.12, 0.025, 0.025]),
                         5: ("arrow(45)", [0.795, 0.075, 0.025, 0.025]), 6: ("line(45)", [0.0624, 0.5655, 0.45, 0.45]),
                         7: ("line(25)", [0.115, 0.453, 0.348, 0.348]), 8: ("line(155)", [0.562, 0.5328, 0.2, 0.2]),
                         9: ("line(30)", [0.557, 0.372, 0.21, 0.21])}
    for _, content in widget_image_dict.items():
        case.set_image(image_path=temp_parent_path + content[0] + ".png", locations=content[1], transparent=True)
    # set texts
    case.set_text(annotation="Membrane", locations=[0.05, 0.35, 0.9, 0.05], backgroundcolor="#FEF4E8",
                  positions=(0.5, 0.3), font_size=12)
    case.set_text(annotation="N-glycan", locations=[0.455, 0.82, 0.04, 0.02], font_size=9, weight="bold",
                  color="#A15153", transparent=True)
    case.set_text(annotation="N-glycan", locations=[0.455, 0.49, 0.04, 0.02], font_size=9, weight="bold",
                  color="#3892F3", transparent=True)
    text_dict1 = {1: ("N-glycan", [0.1, 0.575, 0.05, 0.05], "A15153"), 2: ("SV2A", [0.2, 0.425, 0.05, 0.05], "E59747"),
                  3: (r"/ $H_c$A", [0.35, 0.28, 0.04, 0.03], "999999"), 4: ("BoNT/A", [0.8, 0.9, 0.05, 0.05], "000000"),
                  5: ("115°", [0.51, 0.11, 0.04, 0.04], "000000"), 6: (r"/ $H_c$A", [0.75, 0.28, 0.04, 0.03], "999999"),
                  7: (r"$H_c$E", [0.7, 0.28, 0.04, 0.03], "00CD00"), 8: ("BoNT/E", [0.15, 0.9, 0.05, 0.05], "000000"),
                  9: ("GD1a", [0.3, 0.425, 0.05, 0.05], "00C5CD"), 10: (r"$H_c$E", [0.3, 0.28, 0.04, 0.03], "00CD00"),
                  11: ("SV2C", [0.78, 0.425, 0.05, 0.05], "3892F3"), 12: ("GT1b", [0.88, 0.425, 0.05, 0.05], "639592"),
                  13: ("N-glycan", [0.68, 0.555, 0.05, 0.05], "3892F3")}
    for _, content in text_dict1.items():
        case.set_text(annotation=content[0], locations=content[1], font_size=11, weight="bold", color="#" + content[2],
                      transparent=True)
    text_dict2 = {1: ("SV2C", [0.195, 0.24, 0.04, 0.03], "#3892F3"), 2: ("N", [0.13, 0.23, 0.01, 0.05], "#E59747"),
                  3: ("SV2Ac", [0.13, 0.13, 0.02, 0.03], "#E59747"), 4: ("β8", [0.2, 0.075, 0.01, 0.02], "#E59747"),
                  5: ("C", [0.23, 0.095, 0.01, 0.02], "#E59747"), 6: ("GD1a", [0.335, 0.075, 0.02, 0.02], "#00C5CD"),
                  7: ("/GT1b", [0.39, 0.075, 0.02, 0.02], "#639592"), 8: ("SV2C", [0.61, 0.215, 0.02, 0.02], "#3892F3"),
                  9: ("β8", [0.625, 0.105, 0.01, 0.02], "#3892F3"), 10: ("C", [0.685, 0.14, 0.01, 0.02], "#3892F3"),
                  11: ("N", [0.75, 0.15, 0.01, 0.02], "#3892F3"), 12: ("C", [0.745, 0.085, 0.01, 0.02], "#E59747"),
                  13: ("β8", [0.79, 0.07, 0.01, 0.02], "#E59747"), 14: ("SV2Ac", [0.905, 0.14, 0.01, 0.02], "#E59747"),
                  15: ("N", [0.885, 0.215, 0.01, 0.02], "#E59747")}
    for _, content in text_dict2.items():
        case.set_text(annotation=content[0], locations=content[1], font_size=10, color=content[2])
    text_dict3 = {"a": [0.05, 0.95, 0.05, 0.05], "b": [0.05, 0.3, 0.05, 0.05],
                  "c": [0.425, 0.915, 0.02, 0.02], "d": [0.425, 0.635, 0.02, 0.02]}
    for annotation, content in text_dict3.items():
        case.set_text(annotation=annotation, locations=content, weight="bold", transparent=True)
    case.save_figure(save_parent_path + "manuscript_figure.png")


if __name__ == "__main__":
    designed(file_parent_path="./molecule/manuscript/", temp_parent_path="./temp/", save_parent_path="./designed/")
```

Listing 15: Plotting code lines of case 5 (part 2).

8 Usage of PyMOL-PUB surface (GUI)

Based on PyQt5 [27], PyMOL-PUB offers a process-based plotting GUI to replace the cost of programming Python scripts. It enables a broader spectrum of users, notably those without Python programming experience. By running the windows.py, users can start their drawing jobs using the PyMOL-PUB's GUI. We provide a demo video that shows how to use the GUI to create the target figure. Users can follow along with the video to learn the operation of the GUI, or they can refer to the text description provided below.

8.1 Interface logic

To facilitate users in effortlessly constructing intricate publication-quality figures, we offer a process-oriented GUI, akin to software installation, that guides them in creating their desired figures. The logic between interfaces is shown in Figure 14. It is noteworthy that the transition between interfaces incorporates a data verification feature. Kindly ensure to examine the corresponding prompts diligently to guarantee that the output aligns with the expectations.

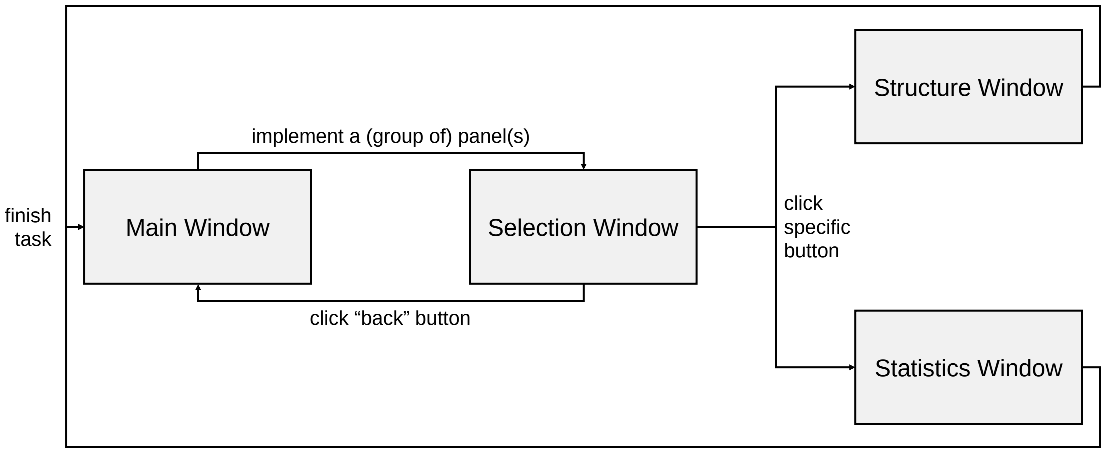

Figure 14: Logic between interfaces

In the Main Window, users can set up the target journal, figure dimensions, and panel layout details by utilizing click or text edit functions. Following the assignment and verification (detailed below) of panel layout information, users can drag the “implement” combo box to designate a panel label, thereby accessing the Selection Window. In the Selection Window, users can choose the content required for the currently selected panel: either structural information or statistical information, and subsequently fulfill the corresponding filling task.

The two tasks adhere to distinct interface-switching logic. For structural information, users follow a 5-step structure exhibition process detailed in the main text to generate structural images. If statistical information is applicable, users can

upload statistical images that they want to paste on the main figure. All operations ultimately revert to the Main Window, where a progress bar below it informs users about the overall panel's actual coverage and task progression. Users can finally output images via the "output" button on the Main Window.

8.2 Main window

The screenshot of the Main Window is depicted in Figure 15. At the top of the interface, a file search button labeled "history" can be found. This term refers to an image or figure that was previously partially finished and now needs to be completed. Upon detection of a history file, this image will first be superimposed onto the output figure, and then the other panel images will be drawn on top. Below this widget are four crucial initialization information combo boxes or text edit boxes. These include the target journal (represented by "targets"), the aspect ratio of the figure (denoted as "height / width"), and the occupation of page width in publications (labeled as "occupied column"). The practical width of the figure is calculated as page width divided by the total column multiplied by the occupied column. The total column (of the page width) combo box becomes usable only after *Cell* is selected in the "targets" combo box.

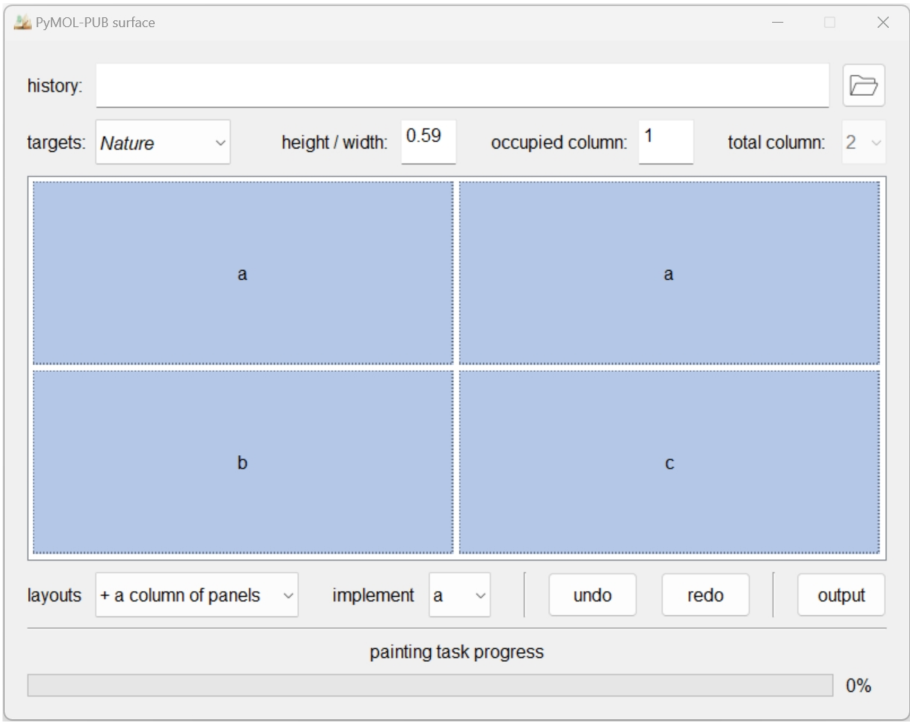

Figure 15: Main window

In the center of the interface, there is a graphic view for marking panel labels, using the pop-up menu as Figure 16. Users can assign a special label in the "label" text edit box to the panel they select, then click the "complete" button to return to the Main Window to complete the panel naming. The layout of the panels can be

adjusted using the commands in the “layouts” combo box to change the number of rows and columns the figure is divided into. On its right side, the “implement” combo box allows for implementing the image drawing of a panel or panel group with the specific panel label. However, if the panel label is set incorrectly, such as having label discontinuity or not meeting rectangular requirements, the “implement” combo box cannot be used. The remaining widgets in the interface consist of three buttons: “undo”, “redo”, and “output”. The first two are completed in the panel implementation dimension and the last one provides the actual production of the expected figure. Lastly, at the bottom of the interface, a progress bar is provided to illustrate the overall actual coverage of the panels, which indicates the task progression situation.

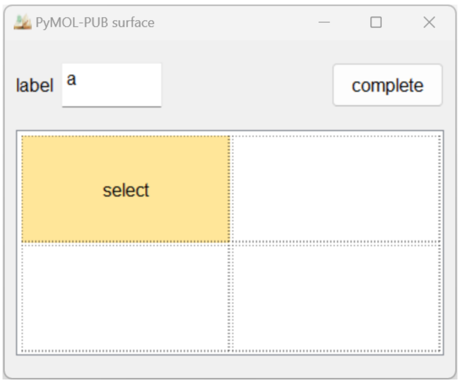

Figure 16: Panel labeling popup menu

8.3 Selection window

As shown in Figure 17, the Selection Window provides two types of content for plotting: structural information and statistical information. Clicking different buttons according to the type of content to be drawn will enter the corresponding drawing process. If users have not yet determined the content to be included in the current panel, they can go back to the Main Window by clicking the “back” button.

8.4 Structure window

The design of the structure image window follows the 5-step exhibition process of structural images, as shown in Figure 4. Therefore, we provide a similar interface skipping logic. A sequential example is shown from Figure 18 to Figure 23.

In the first structure drawing window, as shown in Figure 18 and Figure 19, there are loading and cleaning operations of the structure. At the top of the interface, there is a “path” button for file searching. Clicking the folder button will pop up the computer file browser, where users can select the structure file they want to load. As shown in Figure 18, the protein structure with PDB ID 1F34 is loaded in the window. And there is a “remove” text edit box on the right side of the interface.

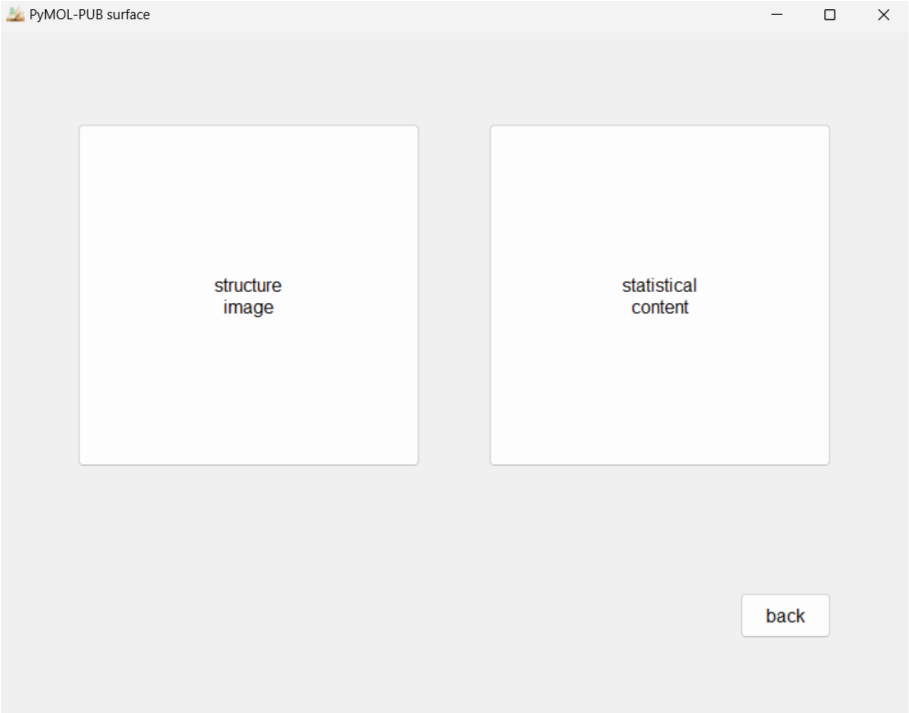

Figure 17: Selection window

Users can enter the structural elements they want to hide to clean up the structural image. As shown in Figure 19, the molecular composition of water in the structure is hidden. The graphical view in the interface is used to display the corresponding image drawn for each step of the operation. In the lower right part of the interface are a series of buttons that respond to different process operations. Each time users click the “refresh” button, the latest image drawn based on the current input parameters will be displayed in the graphical view. Clicking the “undo” button will return to the drawing state of the previous step. Each click will go back one step. When returning to the initial state of the window, “undo” will no longer continue. Clicking the “redo” button will advance to the next drawing state. Each click will advance one step. It will not continue after advancing to the latest drawing state. When clicking the “back” button, users will return to the Selection Window and the operations in the current window will be cleared. When clicking the “next” button, users will enter the next drawing process and experience the new visualization functions.

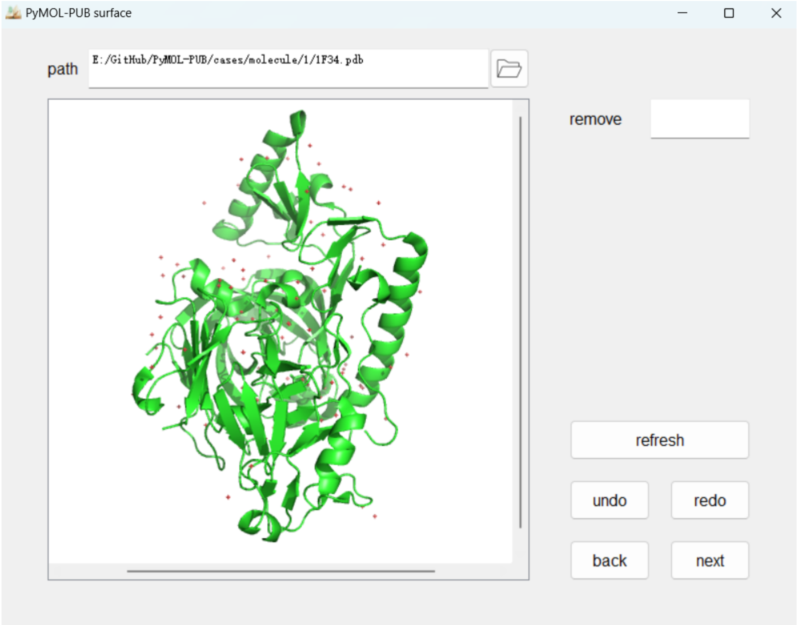

Figure 18: Structure window (operation 1)

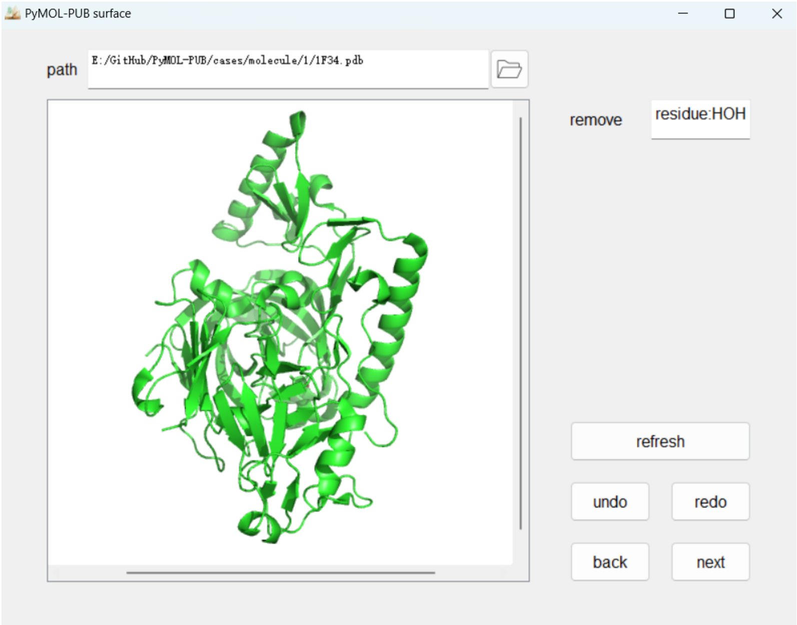

Figure 19: Structure window (operation 2)

In the second structure drawing window, as shown in Figure 20, users can change the representation of the displayed structure. The graphical view in the interface is used to display the corresponding image drawn for each step of the operation. And the two combo boxes and text edit box at the right side of the window can be flexibly adjusted to represent each part of the structure. The specific method of use is to first select a special representation method (such as

“cartoon”, “surface” or “mesh”, etc.) in the “style” combo box, and then select the type of the target element in the “class” combo box (such as “chain”, “model” or “range”, etc.), and finally enter the specific name or number of the target element in the “range” text edit box. As shown in Figure 20, the chain A of structure 1F34 is chosen to be displayed with the “surface” representation. Similarly, in the lower right part of the interface is a series of buttons that respond to different process operations. When clicked, the “back” button will return users to the first structure drawing window.

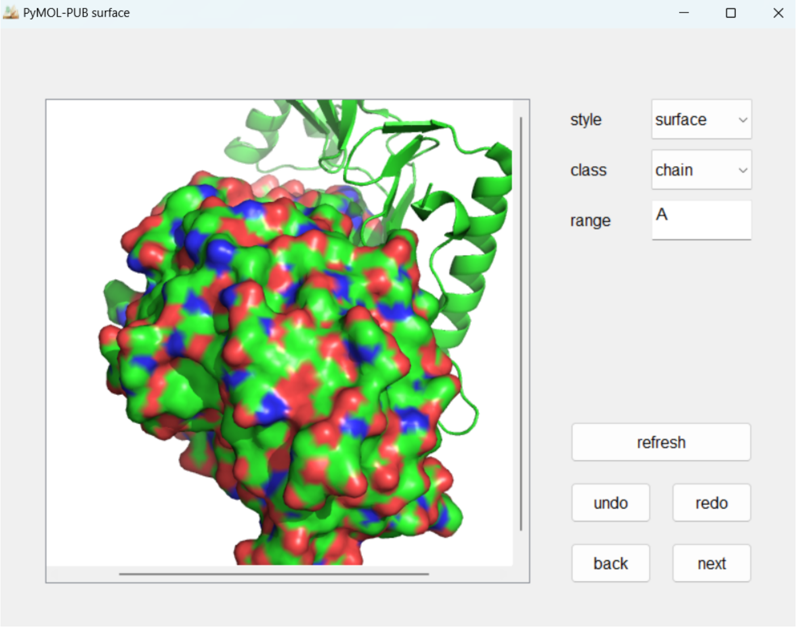

Figure 20: Structure window (operation 3)

In the third structure drawing window, as shown in Figure 21, users can adjust the visual perspective of the structure. The graphic view in the interface is used to display the corresponding image drawn for each step of the operation. And the six text editing boxes on the right side of the window can flexibly adjust the visual perspective of the structure. Here, users rotate the drawing perspective by entering the rotation angle in each dimension into three text edit boxes (labeled as “rotate x”, “rotate y” and “rotate z”). At the same time, users realize the translation of the structure by entering the translation distance in each dimension into three text edit boxes (labeled as “move x”, “move y” and “move z”). As shown in Figure 21, structure 1F34 is rotated 330 degrees on the x-axis, 10 degrees on the y-axis, and 270 degrees on the z-axis. The rotated structural conformation perspective is displayed in the graphical view. Similarly, in the lower right part of the interface is a series of buttons that respond to different process operations. When clicked, the “back” button will take users back to the second structure drawing window.

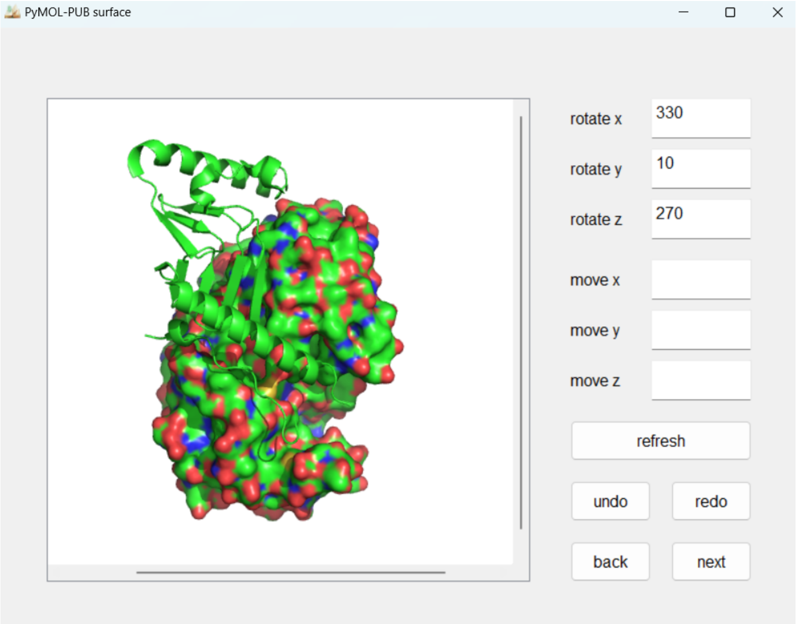

Figure 21: Structure window (operation 4)

In the fourth structure drawing window, as shown in Figure 22 and Figure 23, users can change the display color of the structure. The graphical view in the interface is used to display the corresponding image drawn for each step of the operation. Additionally, the two text edit boxes and combo box on the right side of the window can be used to flexibly adjust the visual color of each part of the structure. The specific method of use is to first enter the specific color name of the target element in the “color” text edit box, and then select the type of the target element in the “class” combo box (such as “chain”, “model” or “range”, etc.), and finally enter the specific name or number of the target element in the “range” text edit box. As shown in Figure 22, we set chain A of structure 1F34 to white (hex number 0xF2F2F2), and as shown in Figure 23, we set chain B of structure 1F34 to blue (hex number 0x2D2F82). Similarly, in the lower right part of the interface, there is a series of buttons that respond to different process operations. When the “back” button is clicked, users will return to the third structure drawing window. When the “next” button is clicked, the image drawing of the currently loaded structure has ended, and users will return to the Main Window to draw a new image or output the target figure.

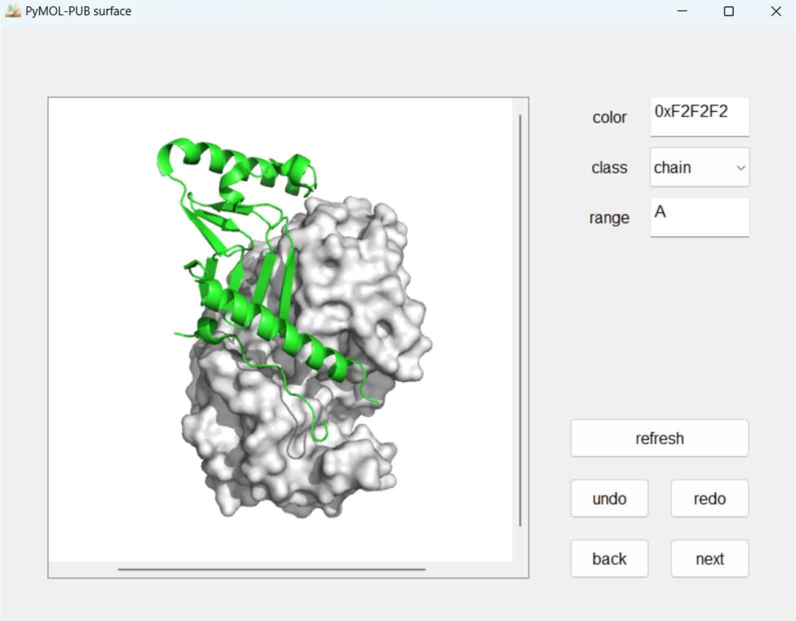

Figure 22: Structure window (operation 5)

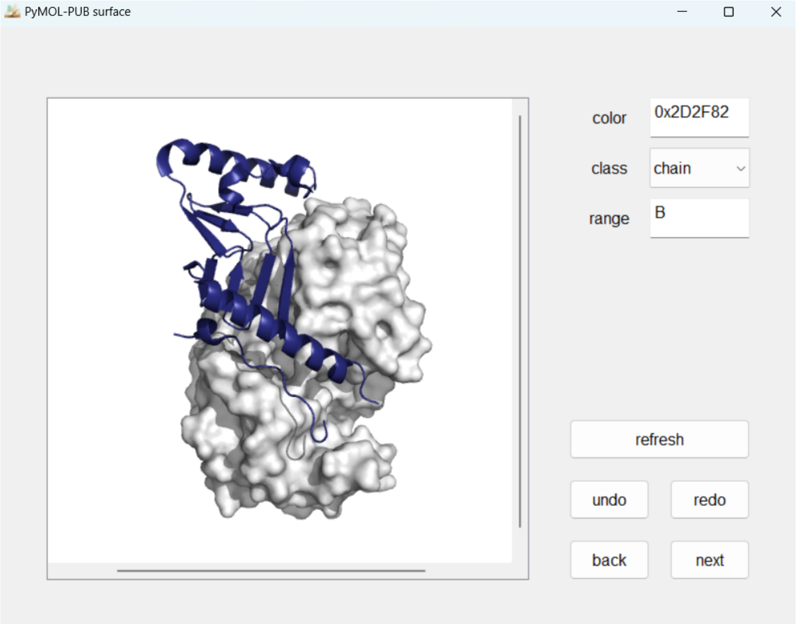

Figure 23: Structure window (operation 6)

8.5 Statistics window

Users can upload statistical images that they want to paste onto the main figure by choosing the Statistics Window, as shown in Figure 24. This window helps users integrate line charts, bar charts, pie charts, and other desired statistical schematics into the target figure.

At the top of the interface, a file search button labeled “path” can be found. Clicking the folder button will pop up the computer files browser, where users can select the image file they want to load. The graphical view in the interface is used to display the corresponding image drawn for each step of the operation. As shown in Figure 24, a statistical image of the chain distribution of the structural data set is loaded in the window. In the lower right part of the interface, there is a series of buttons that respond to different process operations. Each time the user clicks the “refresh” button, the latest image drawn based on the current input parameters will be displayed in the graphical view. Clicking the “undo” button will return to the drawing state of the previous step. Each click will go back one step. When returned to the initial state of the window, “undo” will no longer continue. Clicking the “redo” button will take users to the next drawing state. Each click will advance one step forward. It will not continue after reaching the latest drawing state. When clicking the “back” button, users will return to the Selection Window and the operations in the current window will be cleared. When clicking the “next” button, the pasting of the image to be loaded is complete, and users will return to the Main Window to draw a new image or output the target figure.

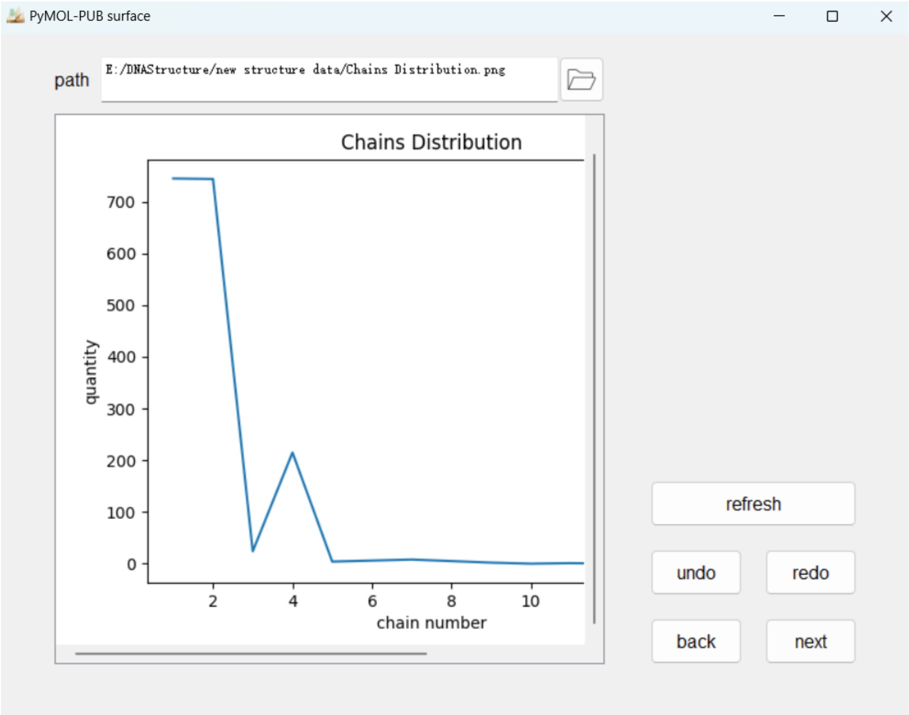

Figure 24: Statistics window

9 References

[1] DeLano, W. L. *et al.* Pymol: An open-source molecular graphics tool. *CCP4 Newsletter on Protein Crystallography* **40**, 82–92 (2002).

[2] Corbi-Verge, C. & Kim, P. M. Motif mediated protein-protein interactions as drug targets. *Cell Communication and Signaling* **14**, 1–12 (2016).

[3] Yang, J. *et al.* Improved protein structure prediction using predicted inter-residue orientations. *Proceedings of the National Academy of Sciences* **117**, 1496–1503 (2020).

[4] Lin, Z. *et al.* Evolutionary-scale prediction of atomic-level protein structure with a language model. *Science* **379**, 1123–1130 (2023).

[5] Zhu, X. *et al.* Structural basis of adhesion gpcr gpr110 activation by stalk peptide and g-proteins coupling. *Nature Communications* **13**, 5513 (2022).

[6] Kabsch, W. A solution for the best rotation to relate two sets of vectors. *Acta Crystallographica Section A: Crystal Physics, Diffraction, Theoretical and General Crystallography* **32**, 922–923 (1976).

[7] Petitjean, M. On the root mean square quantitative chirality and quantitative symmetry measures. *Journal of Mathematical Physics* **40**, 4587–4595 (1999).

[8] Zhang, Y. & Skolnick, J. Tm-align: a protein structure alignment algorithm based on the tm-score. *Nucleic Acids Research* **33**, 2302–2309 (2005).

[9] Zhang, C., Shine, M., Pyle, A. M. & Zhang, Y. Us-align: universal structure alignments of proteins, nucleic acids, and macromolecular complexes. *Nature Methods* **19**, 1109–1115 (2022).

[10] Xu, J. & Zhang, Y. How significant is a protein structure similarity with tm-score= 0.5? *Bioinformatics* **26**, 889–895 (2010).

[11] Mosca, R., Brannetti, B. & Schneider, T. R. Alignment of protein structures in the presence of domain motions. *BMC Bioinformatics* **9**, 1–17 (2008).

[12] Jumper, J. *et al.* Highly accurate protein structure prediction with alphafold. *Nature* **596**, 583–589 (2021).

[13] Frontzek, K. *et al.* A conformational switch controlling the toxicity of the prion protein. *Nature Structural and Molecular Biology* **29**, 831–840 (2022).

[14] Pan, X. *et al.* Molecular basis for pore blockade of human na+ channel nav1. 2 by the μ-conotoxin kiiia. *Science* **363**, 1309–1313 (2019).

[15] Gao, S., Yao, X. & Yan, N. Structure of human cav2. 2 channel blocked by the painkiller ziconotide. *Nature* **596**, 143–147 (2021).

[16] Skolnick, J. & Gao, M. The role of local versus nonlocal physicochemical restraints in determining protein native structure. *Current Opinion in Structural Biology* **68**, 1–8 (2021).

[17] Compeau, P. E., Pevzner, P. A. & Tesler, G. How to apply de bruijn graphs to genome assembly. *Nature Biotechnology* **29**, 987–991 (2011).

[18] Hunter, J. D. Matplotlib: A 2d graphics environment. *Computing in Science and Engineering* **9**, 90–95 (2007).

[19] Huang, F. & Nau, W. M. A conformational flexibility scale for amino acids in peptides. *Angewandte Chemie International Edition* **42**, 2269–2272 (2003).

[20] Tartaglia, G. G. & Vendruscolo, M. Proteome-level interplay between folding and aggregation propensities of proteins. *Journal of molecular biology* **402**, 919–928 (2010).

[21] Cooper, G. & Adams, K. *The cell: a molecular approach* (Oxford University Press, 2023).

[22] Cameselle, J. C., Ribeiro, J. M. & Sillero, A. Derivation and use of a formula to calculate the net charge of acid-base compounds. its application to amino acids, proteins and nucleotides. *Biochemical Education* **14**, 131–136 (1986).

[23] Guckian, K. M. *et al.* Factors contributing to aromatic stacking in water: evaluation in the context of dna. *Journal of the American Chemical Society* **122**, 2213–2222 (2000).

[24] Boldina, G., Ivashchenko, A. & Régnier, M. Using profiles based on nucleotide hydrophobicity to define essential regions for splicing. *International Journal of Biological Sciences* **5**, 13 (2009).

[25] Wetmore, S. D., Boyd, R. J. & Eriksson, L. A. Electron affinities and ionization potentials of nucleotide bases. *Chemical Physics Letters* **322**, 129–135 (2000).

[26] Liu, Z. *et al.* Structural basis for botulinum neurotoxin e recognition of synaptic vesicle protein 2. *Nature Communications* **14**, 2338 (2023).

[27] Meier, B. *Python GUI Programming Cookbook: Develop functional and responsive user interfaces with tkinter and PyQt5* (Packt Publishing Ltd, 2019).
